# Longitudinal microbiome analysis of single donor fecal microbiota transplantation in patients with recurrent *Clostridium difficile* infection and/or ulcerative colitis

**DOI:** 10.1371/journal.pone.0190997

**Published:** 2018-01-31

**Authors:** Michael Mintz, Shanawaj Khair, Suman Grewal, Joseph F. LaComb, Jiyhe Park, Breana Channer, Ramona Rajapakse, Juan Carlos Bucobo, Jonathan M. Buscaglia, Farah Monzur, Anupama Chawla, Jie Yang, Charlie E. Robertson, Daniel N. Frank, Ellen Li

**Affiliations:** 1 Department of Medicine, Stony Brook University, Stony Brook, New York, United States of America; 2 Department of Applied Mathematics and Statistics, Stony Brook University, Stony Brook, New York, United States of America; 3 Department of Pediatrics, Stony Brook University, Stony Brook, New York, United States of America; 4 Department of Family, Population and Preventive Medicine, Stony Brook University, Stony Brook, New York, United States of America; 5 Department of Medicine, University of Colorado Denver, Aurora, Colorado, United States of America; University Hospital Llandough, UNITED KINGDOM

## Abstract

**Background:**

Studies of colonoscopic fecal microbiota transplant (FMT) in patients with recurrent CDI, indicate that this is a very effective treatment for preventing further relapses. In order to provide this service at Stony Brook University Hospital, we initiated an open-label prospective study of single colonoscopic FMT among patients with ≥ 2 recurrences of CDI, with the intention of monitoring microbial composition in the recipient before and after FMT, as compared with their respective donor. We also initiated a concurrent open label prospective trial of single colonoscopic FMT of patients with ulcerative colitis (UC) not responsive to therapy, after obtaining an IND permit (IND 15642). To characterize how FMT alters the fecal microbiota in patients with recurrent *Clostridia difficile* infections (CDI) and/or UC, we report the results of a pilot microbiome analysis of 11 recipients with a history of 2 or more recurrences of *C*. *difficile* infections without inflammatory bowel disease (CDI-only), 3 UC recipients with recurrent *C*. *difficile* infections (CDI + UC), and 5 UC recipients without a history of *C*. *difficile* infections (UC-only).

**Method:**

V3V4 Illumina 16S ribosomal RNA (rRNA) gene sequencing was performed on the pre-FMT, 1-week post-FMT, and 3-months post-FMT recipient fecal samples along with those collected from the healthy donors. Fitted linear mixed models were used to examine the effects of Group (CDI-only, CDI + UC, UC-only), timing of FMT (Donor, pre-FMT, 1-week post-FMT, 3-months post-FMT) and first order Group*FMT interactions on the diversity and composition of fecal microbiota. Pairwise comparisons were then carried out on the recipient vs. donor and between the pre-FMT, 1-week post-FMT, and 3-months post-FMT recipient samples within each group.

**Results:**

Significant effects of FMT on overall microbiota composition (e.g., beta diversity) were observed for the CDI-only and CDI + UC groups. Marked decreases in the relative abundances of the strictly anaerobic Bacteroidetes phylum, and two Firmicutes sub-phyla associated with butyrate production (Ruminococcaceae and Lachnospiraceae) were observed between the CDI-only and CDI + UC recipient groups. There were corresponding increases in the microaerophilic Proteobacteria phylum and the Firmicutes/Bacilli group in the CDI-only and CDI + UC recipient groups. At a more granular level, significant effects of FMT were observed for 81 genus-level operational taxonomic units (OTUs) in at least one of the three recipient groups (p<0.00016 with Bonferroni correction). Pairwise comparisons of the estimated pre-FMT recipient/donor relative abundance ratios identified 6 Gammaproteobacteria OTUs, including the *Escherichia-Shigella* genus, and 2 Fusobacteria OTUs with significantly increased relative abundance in the pre-FMT samples of all three recipient groups (FDR < 0.05), however the magnitude of the fold change was much larger in the CDI-only and CDI + UC recipients than in the UC-only recipients. Depletion of butyrate producing OTUs, such as *Faecalibacterium*, in the CDI-only and CDI + UC recipients, were restored after FMT.

**Conclusion:**

The results from this pilot study suggest that the microbial imbalances in the CDI + UC recipients more closely resemble those of the CDI-only recipients than the UC-only recipients.

## Introduction

In the United States, the incidence of *Clostridium difficile* infection (CDI) has increased considerably in the last two decades, almost tripling between 1996 and 2005 [1, 2]. It is now the leading cause of antibiotic-associated diarrhea. Patients with inflammatory bowel disease (IBD) are at an increased risk for developing CDI [3]. The standard course of treatment for CDI is metronidazole or vancomycin, which have 72% and 79% eradication rates, respectively [4]. However, 20–35% of recovered patients experience a recurrence of CDI, particularly those patients with continued use of antibiotics, use of acid suppression therapy, and older age [5, 6]. Once recurrent CDI occurs, 45–65% of patients will continue to experience recurrent infections over several years [7]. In one study of hospitalized patients, recurrent CDI was associated with higher mortality [8].

Decreased diversity and altered distribution of fecal bacterial taxa was reported in samples collected from patients with recurrent CDI compared to patients with only an initial CDI and patients with non-CDI antibiotic-associated diarrhea [9]. Initial studies indicated that a subset of IBD patients had an altered gut mucosal microbiome compared to non-IBD patients [10]. IBD and CDI were associated with alterations in ileal mucosal microbiome [11]. Twin studies of fecal microbial composition revealed that the microbiota of individuals with CD differed from those of healthy individuals, but were similar between healthy individuals and individuals with ulcerative colitis (UC) [12].

Studies of colonoscopic fecal microbiota transplant (FMT) in patients with recurrent CDI, indicate that this is a very effective treatment for preventing further relapses [13–15]. In order to provide this service at Stony Brook Medicine, we initiated an open-label prospective study of single colonoscopic FMT among patients with ≥ 2 recurrences of CDI, with the intention of monitoring microbial composition in the recipient before and after FMT, as compared with their respective donor. Each of the transplants was conducted using a single known donor, as suggested by the US Food and Drug Association (FDA), to minimize the transmission of occult pathogens (https://www.fda.gov/downloads/biologicsbloodvaccines/guidancecomplianceregulatoryinformation/guidances/vaccines/ucm488223.pdf). Although the FDA has classified human stool as a biological agent, no investigational new drug (IND) permit was required for performing FMT for recurrent CDI. We also initiated a concurrent pilot open label prospective trial of single colonoscopic FMT of patients with UC not responsive to therapy, after obtaining an IND permit (IND 15642). We report here an interim 16S ribosomal RNA (rRNA) sequence analysis of stool samples collected from 19 recipients before, 1 week (wk.) after, and 3 months (mos.) after FMT, as well as the transplant stool samples from their respective donors, who were recruited between December 12, 2013 and the last patient included in this report, completed 1-year follow-up June 14, 2017.

## Materials and methods

### Study protocol

**Fig 1:**
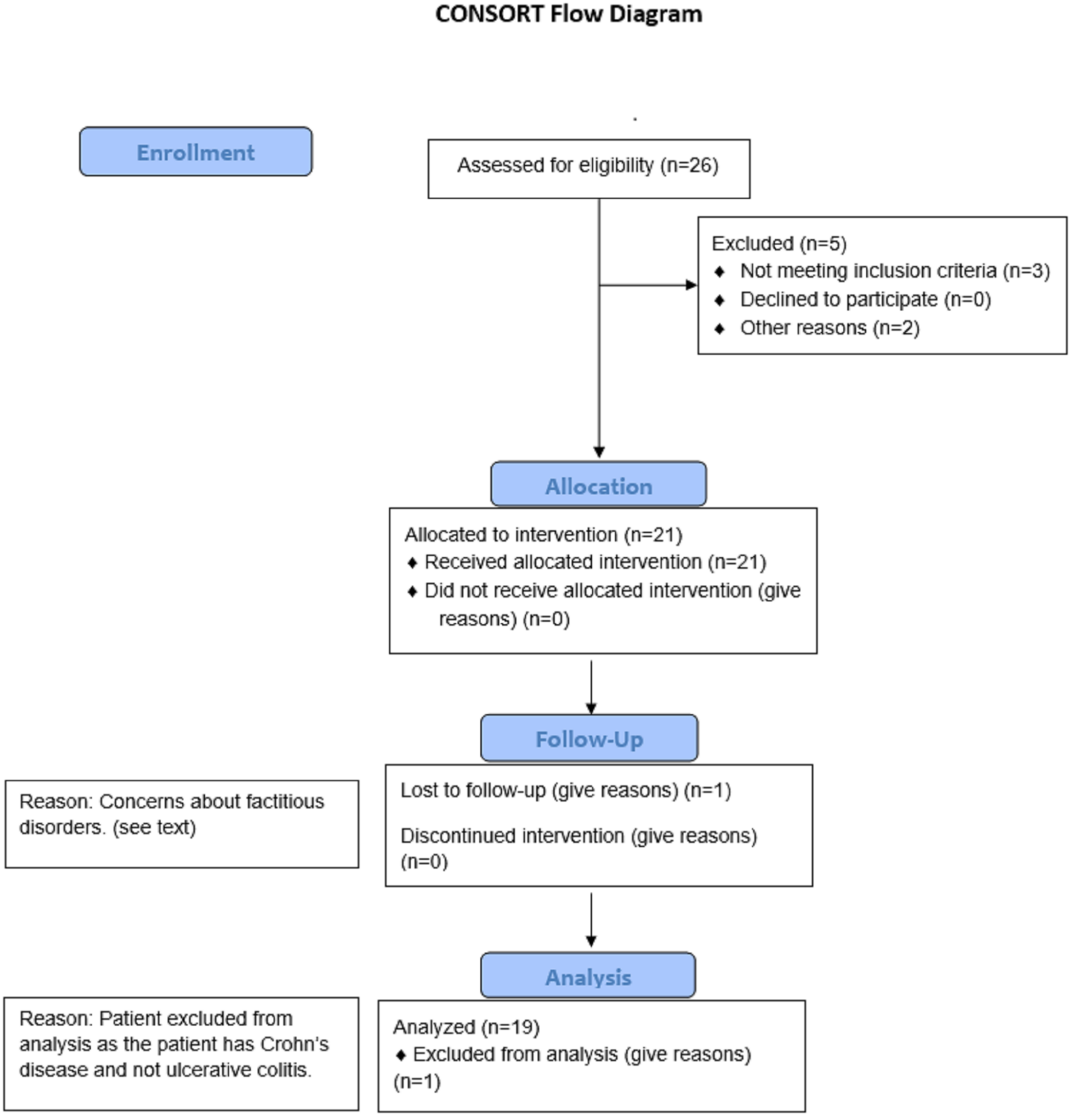
CONSORT flow diagram.

This prospective open label study protocol (see S1 Text. Institutional Review Board (IRB)-approved trial study protocol.) was approved by the Stony Brook University Institutional Review Board (479696) on 11/14/2013 and was registered in ClinicalTrials.gov (Number: NCT03268213, S1 Table. Transparent Reporting of Evaluations with Nonrandomized Designs (TREND) checklist.). The pilot study was started with the plan to enroll subjects from our medical center. However we decided to register as a clinical trial at a later date to enhance enrollment and power of the study. The reason we did not hold recruitment was because this study was designed as a longitudinal observational trial in which all enrolled subject received a fecal microbial transplant from a healthy donor selected by the subject, where each enrolled subject would serve as their own control. We were mistakenly under the impression that only trials where subjects would be randomized to alternative therapies, would require completion of registration prior to enrolling the first patient. For multiple reasons there were delays in completing the PI’s responses to the registry’s queries, although completing these responses did not in any way alter the design of this observational study from that in place prior to registering as a clinical trial. The initial recipient/donor pair was recruited on December 13, 2013. The final recipient included in this report, completed one-year follow up on June 14, 2017. For the patients with recurrent CDI (with or without IBD), the inclusion criteria were ≥ 2 recurrences despite treatment with antibiotics, documented by ≥3 positive stool tests for CDI. For the patients with UC without a history of recurrent CDI, the inclusion criteria were medication refractory UC, requiring step up therapy beyond mesalamine alone. Exclusion criteria for all of the recipients included: a) scheduled for abdominal surgery within the next 12 wks., b) pregnancy, c) Grade 4 anemia (Hemoglobin < 6 g/dL), d) Grade 1 neutropenia (Absolute Neutrophil Count <1500), e) known diagnosis of graft vs. host disease, f) major abdominal surgery within the past 3 mos., g) administration of any investigational drug within the past 2 mos., h) use of a TNF-α antagonist within 2 wks. of the proposed date of transplantation, i) bacteremia within past 4 wks. (28 days), j.) For adults ≥18 years inability to give informed consent. Twenty-six prospective FMT recipients were referred by community physicians, and evaluated and consented by the principal investigator and study coordinator in the Stony Brook University outpatient gastroenterology clinic between November 2013 and June 2016. Five patients were excluded from enrollment (Fig 1). Three of five were excluded, because we could not document ≥ 2 CDI recurrences. The remaining two had not undergone a vancomycin taper for recurrent CDI, and experienced no recurrence after completing the vancomycin taper regimen. Although the protocol was approved for children < 17 y, thus far all of the FMT recipients enrolled in this study have been over the age of 18. One patient was excluded from this report because of concerns about factitious disorder. A second patient with ileal Crohn’s disease and recurrent CDI, recruited during this study period, was excluded from this report, and the results will be reported at a later time, after analysis of additional recipients with Crohn’s disease with and without recurrent CDI. We report here an interim 16S rRNA sequence analysis of stool samples collected before, 1 wk. after, and 3 mos. after FMT, from 19 recipients with recurrent CDI with and without UC and their respective healthy donors, who were recruited between December 12, 2013 and June 2016. The last patient included in this report, completed 1-year follow-up June 14, 2017.

#### FMT donor screening

Each FMT recipient was asked to identify a potential donor, including spouses, parents, family members, friends, or associates of the recipient. The donors were consented and subjected to an initial questionnaire to exclude anyone with: a) known HIV, Hepatitis B, or Hepatitis C infections, b) known exposure to HIV or viral hepatitis (within the previous 12 mos.) c) high-risk sexual behaviors (examples: sexual contact with anyone with HIV/AIDS or hepatitis, sex for drugs or money), d) reported use of illicit drugs within the past 3 mos., e) tattoo or body piercing within the past 6 mos., f) incarceration or history of incarceration, g) known febrile illness within the past 2 wks. of the proposed date of stool donation or current communicable disease (example: upper respiratory tract infection), h) risk factors for variant Creutzfeldt-Jakob disease, i) travel (within the last 3 mos.) to developing countries, j) history of inflammatory bowel disease or chronic diarrhea (i.e. greater than 3 loose stools daily for the past 3 mos.), k) history of gastrointestinal malignancy or known polyposis, l) systemic antibiotics within the preceding 3 mos., m) current use of major immunosuppressive medications (e.g., calcineurin inhibitors, systemic anti-neoplastic, exogenous glucocorticoids, biologic agents), n) any autoimmune disease, moderate to severe malnutrition (BMI<15.0), chronic pain syndrome, metabolic syndrome, or a neurologic or neurodevelopmental disorder, p) atopic disease requiring steroids or immune modulating therapy. Donors were cautioned not to ingest a potential allergen (e.g., nuts) when recipient had known allergy to these agent(s) within 1 wk. prior to the fecal transplant, which would necessitate postponement of the procedure. Potential donors who were not excluded by the screening questionnaire underwent stool testing within 1 mo. of the FMT for: a) *C*. *difficile* toxin B by polymerase chain reaction (PCR); or evaluation for toxins A and B by enzyme immunoassay (EIA), b) bacterial culture for enteric pathogens (*Salmonella*, *Shigella*, *Campylobacter*, *Yersinia*, *Escherichia coli* O157), c) fecal giardia and cryptosporidium antigens, d) acid-fast stains for Cyclospora and Isospora, e) ova and parasites by light microscopy. The donors also underwent serologic testing within 1 mo. stool donation for: a) HIV, type 1 and 2, b) HAV IgM, c) HBsAg, anti-HBc (both IgG and IgM), and anti-HBs, d) HCV Ab, e) RPR and FTA-ABS. Exclusion criteria for donors included age <18, inability to give informed consent, an affirmative response on the above questionnaire, a stool or serologic screening tests, febrile within two wks. of the FMT procedure, ingesting a potential recipient allergen within 1 wk. of the FMT procedure.

#### Preparation of stool transplant

Fresh donor stool was collected the night before or the day of the procedure and stored at 4°C with ice packs provided to the donor. For all cases except one, 50-100g of fresh unfrozen stool was homogenized with a commercial blender in 250–500 ml of sterile saline until it reached a liquid consistency. (In one case, the donor produced only 35g of stool.) The liquid stool was then filtered through gauze pads to remove any particulates and drawn up into 60 ml syringes.

### FMT procedure

To prevent symptomatic CDI recurrence in CDI-only and CDI + UC recipients while they were awaiting transplant, subjects were prescribed a reduced dose of oral vancomycin (usually pulsed dose 125 mg/day 3 x wk.), up until 48 h prior to the procedure. The FMT recipients underwent split dosing polyethylene glycol based preparation the day before the colonoscopy. Approximately 250 ml of the FMT stool filtrate was injected into the terminal ileum/cecum through the biopsy channel by one of four endoscopists. The colonoscopic FMT procedure was performed in the Endoscopy Center at Stony Brook University Hospital. For UC patients, the endoscopist also assessed the appearance of the mucosa to calculate a Mayo endoscopy subscore and this score was utilized to calculate the Mayo score. No biopsies were obtained during the procedure to minimize the risk of complication. The recipients lay on their right side for 1-hour post-procedure in the endoscopy unit prior to discharge.

### Clinical outcomes and follow up

Phone calls were made to the recipient the day after the transplant to determine if there were any immediate adverse events. Recipients then received phone calls once weekly for 12 wks. and then monthly for one year. The caller asked the FMT recipients about the status of their diarrhea (resolved, improved, or worsened), the status of their abdominal pain, the frequency/consistency of their stools, weight changes, changes in medical history/medications, and any other adverse events in general. Adverse events were collected following the NCI Common Terminology Criteria for Adverse Events (CTCAE). For UC patients who underwent a sigmoidoscopy or colonoscopy 3-mos. post-transplant, the Mayo score including the endoscopic subscore was recorded [16]. The patients were also asked if they required any change in UC medication in the 3 mo. follow up period.

### Collection of research stools samples

The FMT recipient research stool samples were collected the day before FMT, 1 wk. after FMT, and 3 mos. after FMT in two specimen containers, one of which contained 10 ml of RNAlater (Qiagen, Germany). The FMT donor specimen was also collected in two specimen containers, one of which contained 10 ml of RNAlater. The specimens were kept cold with ice packs and delivered to the laboratory within 24 h, de-identified and assigned a patient and sample code. The stool samples collected in RNAlater were processed on receipt in the laboratory for extraction of nucleic acids using the Zymo Research DNA MiniPrepTM (Zymo Research Corporation, Irvine, CA) according to the manufacturer’s instructions. The stool samples collected without solution were frozen and stored at -80°C on receipt in the laboratory. The donor and recipients were asked to complete a daily food diary listing what they ate and the serving size during the wk. prior to submitting each stool sample.

### 16S rRNA amplicon library construction

Broad-range PCR amplicons were generated using barcoded primers [17] that target the V3V4 variable region of the 16S rRNA gene: primers 338F (5’ ACTCCTACGGGAGGCAGCAG) and 806R (5’ GGACTACHVGGGTWTCTAAT). PCR products were normalized using a SequalPrepTM kit (Invitrogen, Carlsbad, CA), pooled, lyophilized, purified and concentrated using a DNA Clean and Concentrator Kit (Zymo). Pooled amplicons was quantified using Qubit Fluorometer 2.0 (Invitrogen). The pool was diluted to 4nM and denatured with 0.2 N NaOH at room temperature. The denatured DNA was diluted to 15pM and spiked with 25% of the Illumina PhiX control DNA prior to loading the sequencer. Illumina paired-end sequencing was performed on the Miseq platform with versions v2.4 of the Miseq Control Software and of MiSeq Reporter, using a 600 cycle version 3 reagent kit.

### Analysis of Illumina paired-end reads

Illumina Miseq paired-end reads were aligned to human reference genome hg19 with bowtie2 and matching sequences discarded http://support.illumina.com/sequencing/sequencing_software/igenome.ilmn [18]. All de-multiplexed, paired-end 16S rRNA gene sequence files along with associated metadata were deposited into the NCBI Sequence Read Archive under project number PRJNA412501. The sorted paired reads were assembled using phrap [19, 20]. Pairs that did not assemble were discarded. Assembled sequence ends were trimmed over a moving window of 5 nucleotides (nt) until average quality met or exceeded 20. Trimmed sequences with more than 1 ambiguity or shorter than 350 nt were discarded. Potential chimeras identified with Uchime (usearch6.0.203_i86linux32) [21] using the Schloss [22]. Silva reference sequences were removed from subsequent analyses. Assembled sequences were aligned and classified with SINA (1.3.0-r23838) [23] using the 418,497 bacterial sequences in Silva 115NR99 [24] as reference configured to yield the Silva taxonomy. Operational taxonomic units (OTUs) were produced by clustering sequences with identical taxonomic assignments.

### Statistical analysis

Differences in the characteristics between donor and three recipient groups (recurrent CDI-only, CDI + UC, and UC-only) were compared using Kruskal Wallis with Dunns post-test and chi-square analysis using GraphPAD prism.

Alpha diversity indices (e.g. Chao1, Shannon complexity H, Shannon Evenness H/Hmax) were calculated inferred through 1000 replicate resamplings using Explicet [23], and beta diversity (Bray-Curtis and Jaccard distances) were calculated between the recipient samples and their paired donor samples as previously described [25]. A linear mixed model was used to compare alpha-diversity (ShannonH) and beta-diversity (Bray-Curtis and Jaccard distance) between each timepoint (FMT) and each disease group (Group). The two-way interaction term, Group*FMT, was used to estimate the differences between timepoints within a specific disease group. Unstructured (UN) covariance structure was utilized to model correlation among measurements from the same patient and his/her corresponding donor. P-values less than 0.05 were considered as statistically significant

Because many of the 339 OTUs exhibited zero counts, linear mixed models analyses on individual OTUs at the genus level were conducted on 105 OTUs after eliminating OTUs with an average relative abundance of < 0.001% in the donor and recipient pre-FMT samples, and after discarding OTUs where more than 75% of the samples had a zero count. To compare the relative abundance of each OTU between timepoints before and after FMT [pre-transplant recipient, 1-wk. post-FMT recipient, 3-mos. post-FMT recipient] and each disease group (recurrent CDI-only recipient, CDI + UC recipient, UC-only recipient), a generalized linear mixed model (GLMM) or generalized estimating equation (GEE) were used by taking the actual counts of each OTU as the outcomes that were assumed to follow a negative binomial distribution [25]. The log-transformed overall sequence count for each individual at each timepoint was considered as an offset. Two-way interaction terms (Group*FMT) were used to estimate the difference between the timepoints within a specific disease group. Possible covariance structures to model correlation among longitudinal measurement from the same patient and measurement in the corresponding donor were unstructured (UN) and had compound symmetry (CS). In GEE, the dependence structure was chosen based on Quasi Information Criteria (QIC). Pair-wise p-values were based on the T-test for GLMM, and the Z-test for GEE. The p-values were adjusted for multiple comparisons by the Bonferroni correction or by the Benjamin-Hochberg method (FDR < 0.05). All analysis was performed in SAS 9.4 (SAS institute Inc., Cary, NC) and R 3.4.0 (R Foundation for Statistical Computing, Vienna, Austria).

### Fecal calprotectin analysis

Expression levels of the neutrophilic fecal calprotectin protein, a clinical marker of mucosal inflammation, was measured using the PhiCal Test (Calpro AS, Norway) enzyme-linked immunosorbent assay (ELISA) following the manufacturer’s recommended protocol. Analysis was performed for all groups (CDI-only, CDI + UC, UC-only) and timepoints (pre-FMT (donor and recipient), 1-wk. post-FMT, and 3-mos. Post-FMT). Briefly, approximately 100 mg feces was homogenized in extraction buffer (1:50 weight/volume dilution). Sample extracts were then centrifuged and the resulting supernatant was diluted in dilution solution (1:50) and subjected to the ELISA. Absolute calprotectin levels were quantified by applying the sample test values to the standard curve equation and dilution factor(s). The normal calprotectin range was 0–50 μg/g and > 50 μg/g was considered elevated as indicated by the PhiCal Test.

## Results

### Characteristics of FMT donors and recipients

Between December 2013 and June 2016, 12 recurrent CDI without IBD recipients, 3 UC with recurrent CDI, and 5 UC without recurrent CDI entered the study. One of the 12 recipients with recurrent CDI-only had a serious adverse event and was admitted to the hospital with fever and bacteremia 2 days after FMT. However, it was discovered that this recipient had syringes and opiates in her hospital room, raising concerns about possible factitious disorder. Consequently, this donor/recipient pair was excluded from the analysis. The characteristics of the remaining 19 recipients and their corresponding donors are summarized in Table 1. The median ages of the four donor/recipient groups differed significantly (unadjusted p = 0.009) with the recurrent CDI-only group being substantially older (Table 1). The median pre-transplant fecal calprotectin levels in the four groups were significantly different (unadjusted p = 0.0002) with the CDI + UC and UC-only groups exhibiting higher levels. Neither the donors nor the UC recipients without *C*. *difficile* received any antibiotics within 3 mos. of the transplant, whereas the patients with recurrent CDI with or without UC all received antibiotics within 3 mos. of the transplant although antibiotics were stopped 48 h before the FMT. Inspection of the food diaries of all the recipients revealed that none of the participants (donors or recipients) consumed a vegetarian or gluten free diet.

**Table 1:** Characteristics of FMT donors and recipients (CDI-only, CDI + UC, UC-only).

	Donors n = 19	CDI-only n = 11	CDI + UC n = 3	UC-only n = 5
**Age, years (range)**	46y (26-74y)	66y (34-82y)	32y (29-51y)	34y (29-61y)
**Males n (%)**	8 (42%)	4 (36%)	1 (33%)	3 (60%)
**Caucasian n (%)**	18 (95%)	11 (100%)	3 (100%)	5 (100%)
**Current smoking n (%)**	4 (21%)	1 (10%)	0 (0%)	1 (20%)
**Ex-smoker n (%)**	4 (21%)	5 (45%)	1 (33%)	3 (60%)
**Never smoker n(%)**	11 (58%)	5 (45%)	2 (67%)	1 (20%)
**Antibiotics (3 mos.)**	0 (0%)	11 (100%)	3 (100%)	0 (0%)
**BMI kg/m**^**2**^ **(range)**	25 (20–32.8)	29.1 (17.4–31.6)	24.8 (22.7–27.1)	29.1 (23.3–34.9)
**Fecal calprotectin μg/g (range)**	7.3(0–65.1)	42.1(0–219.7)	175.9 (83.9–342.3)	94.9 (90.8–282.7)

The median values and range of values are listed for each group.

The baseline UC clinical characteristics of individual recipients with UC ± CDI are summarized in Table 2. Only one patient had proctitis and the remaining had left sided or extensive disease (beyond the splenic flexure). All of the patients had been on steroids, immunomodulators, or biologics in addition to mesalamine compounds during the course of their disease. The disease duration ranges from a year to 14 years.

**Table 2:** Pre-FMT UC characteristics.

	Recipient ID	UC Duration (years)	UC Extent (E1-E3)	Fecal Calprotectin (μg/g)	Mayo Score (0–12)	Endo Score (0–3)	UC Medications
**CDI + UC n = 3**	FMT02-1R	10	E2	175.9	2	1	steroids, immunomodulator
	FMT11-1R	1	E3	342.3	8	3	anti-TNFα biologic
	FMT18-1R	1	E2	83.9	2	1	5-ASA, immunomodulator
**UC-only n = 5**	FMT01-1R	14	E1	90.8	2	1	steroids, immunomodulator
	FMT03-1R	1	E2	282.7	4	2	anti-TNFα biologic
	FMT07-1R	10	E2	94.5	9	2	5-ASA only (past steroids and anti-TNFα biologic)
	FMT08-1R	3	E3	94.9	5	2	5-ASA, steroids, immunomodulator, Entyvio
	FMT19-1R	7	E2	227.9	6	2	topical steroids

UC duration was the number of years since UC diagnosis. UC extent was disease extent measured using the Montreal scale: E1, proctitis confined to rectum; E2, left sided disease extend beyond rectosigmoid to splenic flexure; E3 extensive disease past splenic flexure. Normal fecal calprotectin levels were ≤ 50 μg/g. The Mayo and Endo scores were assessed by the endoscopist at the time of the colonoscopy that was performed to instill the donor stool. 5-ASA are mesalamine products.

### Analysis of fecal microbiota

We generated 8,314,757 16S rRNA gene sequences from 71 fecal samples (19 donor, 19 recipient pre-FMT, 19 recipient 1-wk. post-FMT and 14 recipient 3-mos. post-FMT) with an average sequence length of 414 nt, following paired-end merging and trimming. The average sequence depth per sample was 117,109 (minimum 41,608 and maximum 316,073). All libraries had a Good’s coverage score ≥ 99.9% at the rarefaction point of 41,000 sequences, indicating that deep sequence coverage of the intestinal microbiome was achieved for each sample. The majority (≥ 75%) of sequences were binned within 6 phyla/subphyla categories, previously used to characterize the ileal mucosal microbiome in surgical resections samples collected from patients with ileal Crohn’s disease, patients with inflammatory colitis only (predominantly UC) and patients without IBD [11].

**Fig 2:**
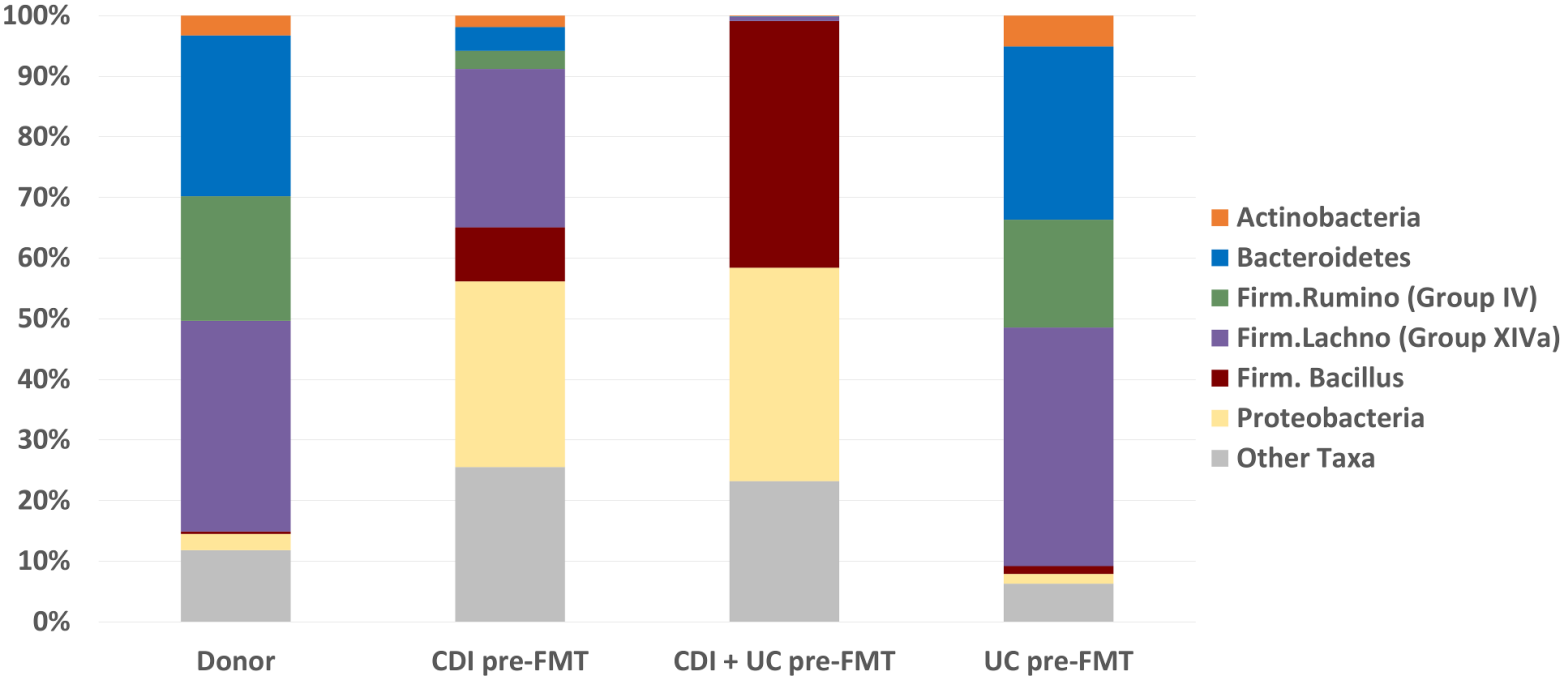
Phyla/subphyla comparison of pre-FMT samples of the CDI-only, UC +CDI, and UC-only recipient groups with the donor samples. The average relative abundance of each of the phyla/subphyla groups is shown for the donor samples and the pre-FMT samples of the CDI-only, CDI + UC and UC-only recipient groups.

The relative abundances of three strictly anaerobic bacterial groups (Bacteroidetes, Firmicutes/Ruminococcaceae and Firmicutes/Lachnospiraceae) were decreased in the CDI-only and CDI + UC recipient pre-FMT fecal samples compared to healthy donors (Fig 2). The relative abundances of two microaerophilic categories (Proteobacteria and Firmicutes/Bacillus) were correspondingly increased in CDI-only and CDI + UC recipient pre-FMT samples compared to healthy donors. These imbalances observed in the CDI-only and CDI + UC recipients are similar to the imbalances previously reported in patients with Crohn’s ileal disease [11]. In contrast, the average relative abundances of these phyla/subphyla categories in pre-FMT UC-only samples were very similar to those of healthy donors.

A fitted linear mixed model was used to measure the effect of patient groups (CDI-only, CDI + UC, UC-only) and FMT (donor, pre-FMT, 1-wk. post-FMT, 3-mos. post-FMT) and first order Group*FMT interactions on alpha diversity as measured by the Shannon H diversity index. P-values were significant for FMT (unadjusted p = 0.0017) and for Group*FMT (unadjusted p = 0.034), but not for Group (unadjusted p = 0.35). Pairwise comparisons of estimated Shannon H differences in alpha-diversity, 95% CI and uncorrected p-values (T-tests) are summarized in Table 3. When the Bonferroni correction for multiple corrections was applied with a threshold of p = 0.0028 (i.e., p = 0.05/18), only the reduced alpha diversity (-1.18, 95% CI -1.80 to -0.56, unadjusted p = 0.0009) between the pre-FMT samples and the 3-mos. post-FMT samples in the CDI + UC group reached statistical significance. The alpha-diversity of the pre-FMT was reduced compared to the 3-mos. post-FMT value (-0.51, 95% CI -.83 to -0.18, unadjusted p = 0.0049) in the CDI-only group, but did not meet the threshold after Bonferroni correction. No significant difference in alpha diversity were observed between the pre-FMT samples and the 3-mos. post-FMT samples (-0.14, 95% CI -0.63 to 0.36, unadjusted p = 0.567) in the UC-only group.

**Table 3:** Estimated differences (Δ) in Shannon H diversity indices between donor and recipient samples within each group.

Group	Comparisons	Δ	95% CI	P-value
**CDI-only n = 11**	Donor vs. Pre-FMT	0.63	(0.20–1.07)	**0.0073**
	Donor vs. 1-wk. post-FMT	0.18	(-0.07–0.44)	0.151
	Donor vs. 3-mos. post-FMT	0.13	(-0.06–0.31)	0.160
	Pre-FMT vs 1-wk. post-FMT	-0.45	(-0.88–0.02)	**0.0416**
	Pre-FMT vs 3-mos. post-FMT	-0.51	(-0.83–0.18)	**0.0049**
	1-wk. post-FMT vs. 3-mos. post-FMT	-0.06	(-0.31–0.20)	0.651
**CDI + UC n = 3**	Donor vs. Pre-FMT	1.02	(0.18–1.85)	**0.0205**
	Donor vs. 1-wk. post-FMT	0.04	(-0.45–0.53)	0.862
	Donor vs. 3-mos. post-FMT	-0.16	(-0.49–0.17)	0.313
	Pre-FMT vs 1-wk. post-FMT	-0.97	(-1.80–0.15)	**0.023**
	Pre-FMT vs 3-mos. post-FMT	-1.18	(-1.80–0.56)	0.0009*
	1-wk. post-FMT vs. 3-mos. post-FMT	-0.20	(-0.68–0.27)	0.375
**UC-only n = 5**	Donor vs. Pre-FMT	0.24	(-0.41–0.89)	0.448
	Donor vs. 1-wk. post-FMT	0.13	(-0.25–0.51)	0.477
	Donor vs. 3-mos. post-FMT	0.10	(-0.18–0.39)	0.459
	Pre-FMT vs 1-wk. post-FMT	-0.11	(-0.74–0.53)	0.728
	Pre-FMT vs 3-mos. post-FMT	-0.14	(-0.63–0.36)	0.567
	1-wk. post-FMT vs. 3-mos. post-FMT	-0.03	(-0.42–0.36)	0.874

Unadjusted p-values are listed with those <0.05 shown in **bold**, while * marks those that are significant following Bonferroni correction.

**Fig 3:**
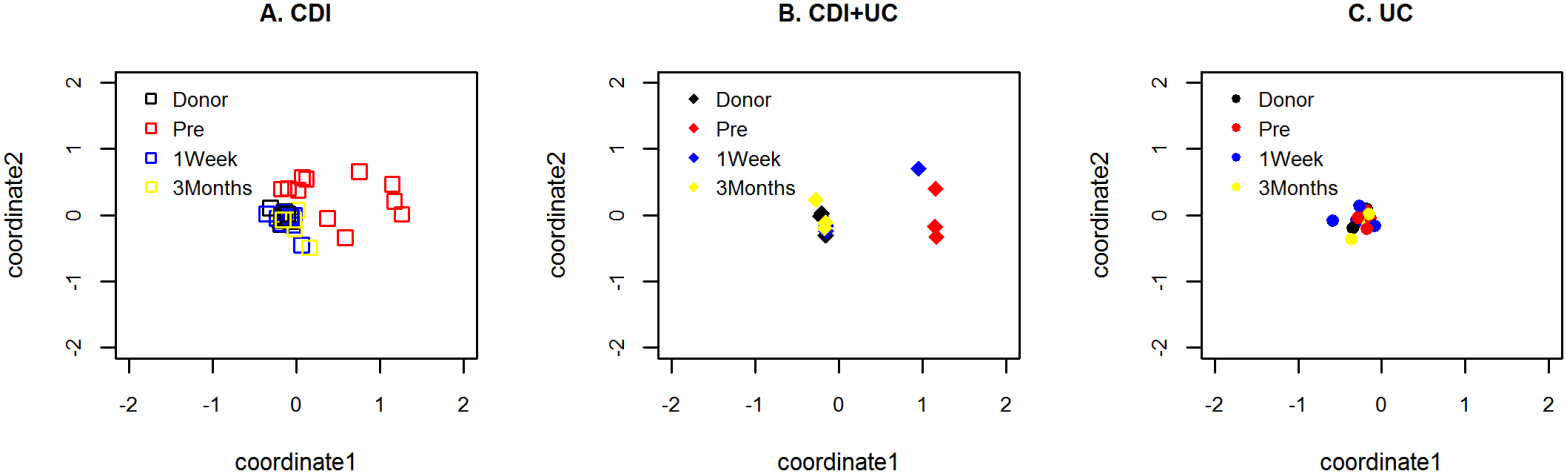
Principal coordinate analysis (PCoA) plots for Bray-Curtis distances (beta-diversity). (A) **CDI-only** recipient group: Donors (**□**), pre-FMT recipient (**□**), 1-wk. post-FMT recipient (**□**), 3-mos. post-FMT recipient (**□**); (B) **CDI + UC** recipient group: Donors (♦), pre-FMT recipient (♦), 1-wk. post-FMT recipient (♦), 3-mos. post-FMT recipient (♦); (C) **UC-only** recipient group: Donors (●), pre-FMT recipient (●), 1-wk. post-FMT recipient (●), 3-mos. post-FMT recipient (●).

To assess overall differences in microbiota composition (i.e. beta-diversity) a fitted linear mixed model was used to measure the effect of patient groups (CDI-only, CDI + UC, UC-only), FMT (pre-FMT, 1-wk. post-FMT, 3-mos. post-FMT), and first order Group*FMT interactions on beta diversity as measured by Bray-Curtis and Jaccard dissimilarities (relative to donor sample). Using Bray-Curtis dissimilarities, the unadjusted p-values were significant for Group (p = 0.009), FMT (p < 0.0001) and Group*FMT (p = 0.004). Similarly, using Jaccard distances, the type 3 values were also significant for Group (p = 0.024), FMT (p = 0.0002) and Group*FMT (p = 0.007). Pairwise comparisons of estimated Bray-Curtis differences in beta-diversity, 95% CI and uncorrected p-values (T-tests) are summarized in Table 4. When the Bonferroni correction for multiple corrections was applied with a threshold of (0.05/9 = 0.0056), both the 1-wk. post-FMT and 3-mos. post-FMT beta-diversity differed significantly from pre-FMT values for the CDI-only and CDI + UC groups, but not for the UC-only group. As illustrated graphically by principle coordinate analysis (PCoA) of the Bray-Curtis indices in Fig 3, FMT appeared to reduce beta diversity differences between the CDI-only and CDI + UC recipients and their donors. Similar results were observed using Jaccard distances.

**Table 4:** Estimated differences (Δ) in Bray Curtis distances for recipient samples relative to donor samples between timepoints for each recipient group (CDI-only, CDI + UC, UC-only).

Groups	Comparisons	Δ	95% CI	P-value
**CDI-only n = 11**	Pre-FMT vs 1-wk. post-FMT	0.41	(0.30–0.52)	<.0001*
	Pre-FMT vs 3-mos. post-FMT	0.449	(0.33–0.57)	<.0001*
	1-wk. post-FMT vs. 3-mos. post-FMT	0.039	(-0.08–0.158)	0.51
**CDI + UC n = 3**	Pre-FMT vs 1-wk. post-FMT	0.33	(0.13–0.54)	0.0026*
	Pre-FMT vs 3-mos. post-FMT	0.50	(0.29–0.70)	<.0001*
	1-wk. post-FMT vs. 3-mos. post-FMT	0.17	(-0.039–0.37)	0.108
**UC-only n = 5**	Pre-FMT vs 1-wk. post-FMT	-0.022	(-0.18–0.14)	0.78
	Pre-FMT vs 3-mos. post-FMT	0.018	(-0.17–0.21)	0.85
	1-wk. post-FMT vs. 3-mos. post-FMT	0.039	(-0.15–0.23)	0.67

The unadjusted p-values are listed and * mark those that are significant by Bonferroni correction.

### Effect of group, FMT, and Group*FMT on individual genus-level OTUs

A fitted linear mixed model was used to measure the effect of patient groups (CDI-only, CDI + UC, UC-only), FMT (donor, pre-FMT, 1-wk. post-FMT, 3-mos. post-FMT), and first order Group*FMT interactions on the relative abundances of individual genus-level OTUs. 81 of 105 OTUs were significantly affected by FMT (p <0.00016 after Bonferroni correction). Pairwise comparisons of the estimated recipient/donor ratios of relative abundances and the estimated recipient ratios of relative abundances between the three timepoints were conducted for each of these OTUs. Seventy-nine OTUs exhibited significant estimated pre-FMT recipient/donor ratios (FDR <0.05) in at least one of the three recipient groups (CDI-only, CDI + UC, and UC-only). In most cases, (OTUs indicated by * in Tables 5–11), also exhibited significant (FDR < 0.05) estimated 1-wk. post-FMT/pre-FMT ratios and/or 3-mos. post-FMT ratios of relative abundances.

**Table 5:** The estimated pre-FMT recipient/donor ratios of the relative abundances of Actinobacteria OTUs for each recipient group.

Actinobacteria OTU	CDI	CDI + UC	UC
*Actinomycetales/Actinomycetaceae/Actinomyces*		3.1* (2–4.9)	
*Bifidobacteriales/Bifidobacteriaceae/Bifidobacterium*	8.9 * (1.7–46.1)	0.023* (0.003–0.15)	3.66 (2.4–5.6)
*Coriobacteriia/Coriobacteriales/Coriobacteriaceae*	0.02* (0.005–0.08)	0.001* (0–0.008)	
*Coriobacteriia/Coriobacteriales/Coriobacteriaceae/Collinsella*	0.002* (0–0.009)	0.001* (0–0.005)	
*Coriobacteriia/Coriobacteriales/Coriobacteriaceae/Gordonibacter*		0.019* (0.016–0.023)	

A ratio > 1 indicated ↑ relative abundance in recipient pre-FMT compared to donor samples. A ratio of < 1 indicated ↓ relative abundance in pre-FMT samples compared to donor samples. The 95% confidence levels are shown in parentheses. Only the estimated ratios with an FDR < 0.05 are listed. The * indicates those OTUs that also had a significant estimated ratios of post-FMT/pre-FMT relative abundances at the one week and/or 3 month time point, indicating that the FMT had a significant effect.

**Table 6:** The estimated pre-FMT recipient/donor ratios of the relative abundances of Bacteroidetes OTUs for each recipient group.

Bacteroidetes OTU	CDI	CDI + UC	UC
*Bacteroidia/Bacteroidales/Bacteroidaceae/Bacteroides*	0.16* (0.06–0.48)	0.001* (0.001–0.003)	1.81 (1.12–2.92)
*Bacteroidia/Bacteroidales/Prevotellaceae*		0.33 (0.17–0.64)	122* (27.97–531.7)
*Bacteroidia/Bacteroidales/Porphyromonadaceae/Barnesiella*	0.006* (0.002–0.016)	0.004* (0–0.036)	8.86* (3.88–20.27)
*Bacteroidia/Bacteroidales/Porphyromonadaceae/Butyricimonas*	0.016* (0.009–0.026)		
*Bacteroidia/Bacteroidales/Porphyromonadaceae/Odoribacter*	0.001* (0–0.004)	0* (0–0.002)	
*Bacteroidia/Bacteroidales/Porphyromonadaceae/Parabacteroides*	0.024* (0.005–0.116)	0.003* (0.002–0.004)	
*Bacteroidia/Bacteroidales/Rikenellaceae/Alistipes*	0.001* (0.001–0.003)	0* (0–0.002)	
*Bacteroidia/Bacteroidales/S24-7*		0.123* (0.05–0.301)	0.579 (0.492–0.682)
*VC2*.*1-Bac22*	0.138* (0.05–0.383)	0.374 (0.201–0.696)	
*Unassigned*	0.007* (0.001–0.038)	0.095* (0.042–0.218)	0.052 (0.005–0.52)

A ratio > 1 indicated ↑ relative abundance in recipient pre-FMT compared to donor samples. A ratio of < 1 indicated ↓ relative abundance in pre-FMT samples compared to donor samples. The 95% confidence levels are shown in parentheses. Only the estimated ratios with an FDR < 0.05 are listed. The * indicates those OTUs that also had a significant estimated ratios of post-FMT/pre-FMT relative abundances at the one week and/or 3 month time point, indicating that the FMT had a significant effect.

**Table 7:** The estimated pre-FMT recipient/donor ratios of the relative abundances of Firmicutes.Ruminococcaceae (Clostridia Group IV) OTUs for each recipient Group.

Firmicutes.Ruminococcaceae OTU	CDI	CDI + UC	UC
*Clostridiales/Ruminococcaceae/Anaerofilum*	0.082* (0.046–0.149)		
*Clostridiales/Ruminococcaceae/Anaerotruncus*		0* (0, 0)	
*Clostridiales/Ruminococcaceae/Faecalibacterium*	0.004* (0.002–0.007)	0.003* (0.001–0.008)	
*Clostridiales/Ruminococcaceae/Hydrogenoanaerobacterium*	11.1* (1.75–70.88)	0.346 (0.143–0.841)	
*Clostridiales/Ruminococcaceae/Oscillospira*	0.07* (0.015–0.329)		9.69 (1.87–50.25)
*Clostridiales/Ruminococcaceae/Ruminococcus*	0.053 (0.008–0.36)	0.011* (0.004–0.026)	0.484* (0.391–0.601)
*Clostridiales/Ruminococcaceae/Subdoligranulum*	0.023* (0.003–0.156)	0.002* (0.001–0.007)	
*Clostridiales/Ruminococcaceae*	0.216* (0.079–0.587)	0.001* (0–0.002)	

A ratio > 1 indicated ↑ relative abundance in recipient pre-FMT compared to donor samples. A ratio of < 1 indicated ↓ relative abundance in pre-FMT samples compared to donor samples. The 95% confidence levels are shown in parentheses. Only the estimated ratios with an FDR < 0.05 are listed. The * indicates those OTUs that also had a significant estimated ratios of post-FMT/pre-FMT relative abundances at the one week and/or 3 month time point, indicating that the FMT had a significant effect.

**Table 8:** The estimated ratios (pre-FMT recipient/donor) of the relative abundances of Firmicutes.Lachnospiraceae (Clostridia GroupXIVa) OTUs for each recipient group.

Firmicutes.Lachnospiraceae OTU	CDI	CDI + UC	UC
*Clostridiales/Lachnospiraceae/Anaerostipes*		0.002* (0.001–0.004)	
*Clostridiales/Lachnospiraceae/Blautia*		0.001* (0.001–0.002)	
*Clostridiales/Lachnospiraceae/Coprococcus*		0.001* (0–0.003)	
*Clostridiales/Lachnospiraceae/Dorea*	0.006* (0.002–0.015)	0.004* (0.001–0.011)	
*Clostridiales/Lachnospiraceae/Lachnospira*		0.001* (0–0.016)	
*Clostridiales/Lachnospiraceae/Marvinbryantia*		0.061* (0.01, 0.362)	
*Clostridiales/Lachnospiraceae/Pseudobutyrivibrio*	0.042* (0.008–0.227)	0.001* (0–0.003)	
*Clostridiales/Lachnospiraceae/Roseburia*		0.001* (0–0.002)	0.566 (0.403–0.795)
*Clostridiales/Lachnospiraceae*		0.045* (0.017–0.116)	

A ratio > 1 indicated ↑ relative abundance in recipient pre-FMT compared to donor samples. A ratio of < 1 indicated ↓ relative abundance in pre-FMT samples compared to donor samples. The 95% confidence levels are shown in parentheses. Only the estimated ratios with an FDR < 0.05 are listed. The * indicates those OTUs that also had a significant estimated ratios of post-FMT/pre-FMT relative abundances at the one week and/or 3 month time point, indicating that the FMT had a significant effect.

**Table 9:** The estimated pre-FMT recipient/donor ratios of the relative abundances of Firmicutes.Bacilli OTUs for each recipient group.

Firmicutes.Bacilli OTU	CDI	CDI + UC	UC
*Bacillales/Planococcaceae/Planomicrobium*	14.91* (4.66–47.75)	25.61* (11.53–56.94)	
*Bacillales/Staphylococcaceae/Staphylococcus*	3.54* (1.46–8.56)	0.492 (0.303–0.798)	21.41* (4.27–107.45)
*Lactobacillales*	56.37* (15.49–205.00)	44.12* (9.14–212.94)	
*Lactobacillales/Carnobacteriaceae*	24.31* (6.30–93.79)	7.52* (1.99–28.45)	
*Lactobacillales/Enterococcaceae/Enterococcus*	21.85* (3.15–151.56)		33.85* (11.39–100.59)
*Lactobacillales/Lactobacillaceae/Lactobacillus*	181.82* (34.50–958.1)	1806.24* (1208.34–2697.28)	122.85* (27.39–551.15)
*Lactobacillales/Streptococcaceae*	4.75* (1.44–15.72)	7.26* (4.07–12.95)	
*Lactobacillales/Streptococcaceae/Lactococcus*		0.49* (0.42–0.57)	14.64* (3.29–65.30)
*Lactobacillales/Streptococcaceae/Streptococcus*	5.35* (1.93–14.85)	17.73 (7.37–42.65)	3.20 (1.31–7.77)
*Unassigned*	96.35* (22.04–421.16)	61.62* (20.11–188.67)	

A ratio > 1 indicated ↑ relative abundance in recipient pre-FMT compared to donor samples. A ratio of < 1 indicated ↓ relative abundance in pre-FMT samples compared to donor samples. The 95% confidence levels are shown in parentheses. Only the estimated ratios with an FDR < 0.05 are listed. The * indicates those OTUs that also had a significant estimated ratios of post-FMT/pre-FMT relative abundances at the one week and/or 3 month time point, indicating that the FMT had a significant effect.

**Table 10:** The estimated pre-FMT recipient/donor ratios of the relative abundances of Proteobacteria OTUs for each recipient group.

Proteobacteria OTU	CDI	CDI + UC	UC
*Alphaproteobacteria/Rhodospirillales/Rhodospirillaceae/Thalassospira*	0.004* (0.001–0.012)	0.003* (0.001–0.008)	0.193* (0.189–0.197)
*Betaproteobacteria/Burkholderiales/Alcaligenaceae/Sutterella*		0.002* (0.001–0.005)	
*Deltaproteobacteria/Desulfovibrionales/Desulfovibrionaceae/Bilophila*		0.004* (0.002–0.009)	
*Gammaproteobacteria/B38*	12.42* (4.51–34.16)	24.12* (7.51–77.40)	
*Gammaproteobacteria/Enterobacteriales/Enterobacteriaceae*	55.81* (6.55–475.80)	1605.19* (287.15–8982.2)	13.38* (3.06–58.62)
*Gammaproteobacteria/Enterobacteriales/Enterobacteriaceae/Citrobacter*	48.09* (7.71–299.76)	2162.46* (511.83–9145.34)	2.33* (1.54–3.52)
*Gammaproteobacteria/Enterobacteriales/Enterobacteriaceae/Enterobacter*	252.65* (90.47–704.86)	167.00* (60.40–461.28)	
*Gammaproteobacteria/Enterobacteriales/Enterobacteriaceae/Escherichia-Shigella*	13.61* (2.82–65.76)	23.64* (5.74–97.42)	10.31 (3.76–28.25)
*Gammaproteobacteria/Enterobacteriales/Enterobacteriaceae/Klebsiella*	1998.2* (278.4–14342.8)	358.89* (87.36–1475.87)	24.24* (3.39–173.12)
*Gammaproteobacteria/Enterobacteriales/Enterobacteriaceae/Proteus*	1384.37* (353.90–5409.98)	1560.87* (205.00–11896.51)	38.59* (8.52–174.86)
*Gammaproteobacteria/Unassigned*	107.45* (17.57–657.87)	165.34* (41.47–659.18)	34.85* (4.15–292.66)
*Unassigned*	12.88* (5.85–28.33)	6.10 (3.58–10.38)	3.60* (1.55–8.37)

A ratio > 1 indicated ↑ relative abundance in recipient pre-FMT compared to donor samples. A ratio of < 1 indicated ↓ relative abundance in pre-FMT samples compared to donor samples. The 95% confidence levels are shown in parentheses. Only the estimated ratios with an FDR < 0.05 are listed. The * indicates those OTUs that also had a significant estimated ratios of post-FMT/pre-FMT relative abundances at the one week and/or 3 month time point, indicating that the FMT had a significant effect.

**Table 11:** The estimated pre-FMT recipient/donor ratios of the relative abundances of OTUs in “Other Taxa” for each recipient group.

Other Firmicutes OTU	CDI	CDI + UC	UC
*Clostridia/Clostridiales/Christensenellaceae*	0.087* (0.014–0.544)	0.001* (0.001–0.002)	
*Clostridia/Clostridiales/Clostridiaceae*		0.095* (0.041–0.222)	
*Clostridia/Clostridiales/Eubacteriaceae/Anaerofustis*		0.108* (0.021–0.549)	
*Firmicutes/Clostridia/Clostridiales/Eubacteriaceae/Eubacterium*	0.154* (0.053–0.443)	0.927 (0.896–0.959)	
*Clostridia/Clostridiales/Family-XI-Incertae-Sedis/Anaerococcus*		0.25* (0.095–0.658)	33.05* (6.56–166.50)
*Clostridia/Clostridiales/Family-XI-Incertae-Sedis/Parvimonas*			7.029* (5.296–9.328)
*Clostridia/Clostridiales/Family-XI-Incertae-Sedis/Peptoniphilus*			11.001 (3.347–36.162)
*Clostridia/Clostridiales/Family-XIII-Incertae-Sedis*		0.005* (0.001–0.021)	
*Clostridia/Clostridiales/Peptococcaceae*	0.054* (0.029–0.104)	0.009* (0.001–0.061)	
*Clostridia/Clostridiales/Peptococcaceae/Peptococcus*	0.026* (0.013–0.051)	0.925* (0.898–0.953)	
*Clostridia/Clostridiales/Peptostreptococcaceae*		0.088 (0.053–0.148)	
*Erysipelotrichia/Erysipelotrichales/Erysipelotrichaceae/Catenibacterium*	0.074* (0.013–0.414)	0.009* (0.001–0.095)	
*Erysipelotrichi/Erysipelotrichales/Erysipelotrichaceae/Turicibacter*		0.149* (0.029, 0.757)	
*Negativicutes/Selenomonadales/Acidaminococcaceae/Acidaminococcus*		0.084* (0.013–0.538)	0.004* (0.002–0.012)
*Negativicutes/Selenomonadales/Acidaminococcaceae/Phascolarctobacterium*		0.009* (0.001–0.065)	
*Negativicutes/Selenomonadales/Veillonellaceae*	1605.19* (492.75–5229.13)	1055.7* (758.2–1468.5)	
*Negativicutes/Selenomonadales/Veillonellaceae/Dialister*		0.009* (0.004–0.02)	
*Negativicutes/Selenomonadales/Veillonellaceae/Megamonas*	0.023* (0.006–0.087)		0.001* (0–0.021)
*Negativicutes/Selenomonadales/Veillonellaceae/Megasphaera*		34.71* (2.38–506.23)	0.004* (0.002–0.01)
*Negativicutes/Selenomonadales/Veillonellaceae/Veillonella*	385.29* (145.04–1023.52)	559.48* (221.19–1416.58)	
**Fusobacteria OTU**	**CDI**	**CDI + UC**	**UC**
*Fusobacteriia/Fusobacteriales*	147.23* (39.17–552.80)	837.99* (137.14–5125.59)	2.44 (1.12–5.29)
*Fusobacteriia/Fusobacteriales/Fusobacteriaceae/Fusobacterium*	4363.4* (1246.4–15290.7)	3487.7* (857.5–14200.0)	34.74* (31.82–37.94)
**Candidate-division-TM7 OTU**			
*Candidate-division-TM7*		9.92* (5.88–16.76)	
**Verrucomicrobia OTU**			
*Verrucomicrobiae/Verrucomicrobiales/Verrucomicrobiaceae/Akkermansia*	9.34* (3.25–26.79)		
*Bacteria/Unassigned*	0.167* (0.086–0.322)	0.032* (0.02–0.051)	

A ratio > 1 indicated ↑ relative abundance in recipient pre-FMT compared to donor samples. A ratio of < 1 indicated ↓ relative abundance in pre-FMT samples compared to donor samples. The 95% confidence levels are shown in parentheses. Only the estimated ratios with an FDR < 0.05 are listed. The * indicates those OTUs that also had a significant estimated ratios of post-FMT/pre-FMT relative abundances at the one week and/or 3 month time point, indicating that the FMT had a significant effect.

### Quantitative PCR assay of *F*. *prausnitzii* relative abundance

Targeted PCR analysis of the relative abundance of *F*. *prausnitzii* was conducted to confirm the results for the *Faecalibacterium* genus. A fitted linear mixed model was used to measure the effect of patient group (CDI-only, CDI + UC, UC-only) and FMT (donor, pre-FMT, 1-wk. post-FMT, 3-mos. post-FMT) and the interaction term on log_2_ (*F*. *prausnitzii/* total bacteria). The unadjusted p values were significant for FMT (p<0.0001) and Group*FMT (p = 0.008), but did not reach significance for group (p = 0.054). Pairwise comparisons of the estimated log_2_ (*F*. *prausnitzii*/ total bacteria) are summarized in Table 12. Pre-FMT samples had reduced relative abundance of *F*. *prausnitzii* compared to the donor in both CDI-only patients (-9.36, 95% CI -11.88 to -6.85, p<0.0001) and CDI + UC patients (-10.3, 95% CI -15.08 to -5.52, p = 0.0003), but not in UC-only patients (-1.52, 95% CI -5.18 to 2.15, p = 0.396). Following transplant, only the CDI-only patients experienced an increase in relative abundance of *F*. *prausnitzii*. These patients had increased relative abundance at both 1-wk. (8.98, 95% CI 5.28 to 12.69, p<0.0001) and 3-mos., (9.04, 95% CI 4.65 to 13.43, p = 0.0004) post-FMT. Patients with CDI + UC showed a trend of increasing relative abundance 3-mos. post-transplant (12.23, 95%CI 4.17 to 20.28, p = 0.005), but did not reach significance with the Bonferroni correction. Patients with UC did not show an increase in their relative abundance of *F*. *prausnitzii* following transplant.

**Table 12:** Estimated differences in log_2_ (*F*. *Prausnitzii/*total bacteria) between donor and recipient samples from each recipient group (CDI-only, CDI + UC, UC-only) and between timepoints.

Group	Comparisons	Δ	95% CI	Unadjusted P-value*
**CDI-only**	Pre-FMT vs Donor	-9.36	(-11.88,-6.85)	<.0001*
	1-wk. post-FMT vs Donor	-0.38	(-3.01, 2.25)	0.7637
	3-mos. post-FMT vs Donor	-0.32	(-3.5, 2.86)	0.8333
	1-wk. post-FMT vs Pre-FMT	8.98	(5.28, 12.69)	<.0001*
	3-mos. post-FMT vs Pre-FMT	9.04	(4.65, 13.43)	0.0004*
	3-mos. post-FMT vs 1-wk. post-FMT	0.06	(-2.83, 2.95)	0.9664
**CDI + UC**	Pre-FMT vs Donor	-10.3	(-15.08, -5.52)	0.0003*
	1-wk. post-FMT vs Donor	-3.43	(-8.44, 1.58)	0.1672
	3-mos. post-FMT vs Donor	1.93	(-3.66, 7.52)	0.4779
	1-wk. post-FMT vs Pre-FMT	6.86	(-0.24, 13.97)	0.0574
	3-mos. post-FMT vs Pre-FMT	12.23	(4.17, 20.28)	0.0051
	3-mos. post-FMT vs 1-wk. post-FMT	5.36	(0.32, 10.4)	0.0382
**UC-only**	Pre-FMT vs Donor	-1.52	(-5.18, 2.15)	0.3964
	1-wk. post-FMT vs Donor	3.41	(-0.47, 7.29)	0.0812
	3-mos. post-FMT vs Donor	2.47	(-1.92, 6.85)	0.2525
	1-wk. post-FMT vs Pre-FMT	4.93	(-0.57, 10.42)	0.0759
	3-mos. post-FMT vs Pre-FMT	3.98	(-2.36, 10.33)	0.2035
	3-mos. post-FMT vs 1-wk. post-FMT	-0.94	(-4.95, 3.07)	0.6276

* Marks those that are significant by Bonferroni correction (p = 0.0028 (0.05/18)).

### CDI recurrence in CDI-only and CDI + UC groups

Of the 11 CDI-only recipients, there were no CDI recurrences after one year of follow up. Of the 3 CDI + UC recipients, one recipient had recurrence of *C*. *difficile* infection 11 mos. after FMT and was treated with another FMT. Of note the relative abundance of *F*. *prausnitzii* in the pre-FMT samples for the second FMT was significantly increased compared to the first FMT (data not shown). Two years after the second FMT, this patient has not had further recurrence of *C*. *difficile* infection.

### Changes in fecal calprotectin levels, Mayo scores, endoscopic scores and medications in individual CDI + UC and UC-only recipients during the 3-mos. post-FMT

None of the CDI + UC or UC-only recipients experienced a serious adverse event during the 3 mos. after transplant. Three recipients were taken off steroids or had reduced their steroid dose. Two recipients underwent no change in medications. One recipient was started on an anti-TNFα biologic. One recipient was switched from an anti-TNFα biologic to Entyvio or vedolizumab. One recipient opted to undergo elective total colectomy with ileostomy. As summarized in Table 13, the range in post-FMT changes in fecal calprotectin levels ranged from a decrease of 49.6 μg/g to an increase of 190.5 μg/g compared to pre-FMT levels. Of the four recipients who underwent a follow-up sigmoidoscopy 3-mos. post-FMT, neither the Mayo nor Endoscopy scores were significantly increased.

**Table 13:** Post FMT changes in UC characteristics. The changes were calculated relative to pre-FMT UC characteristics (see Table 2). N.A. means not available because stool sample was not collected or sigmoidoscopy/colonoscopy was not performed at 3 months.

	Recipient ID	1 wk post-FMT Δ fecal calprotectin μg/g	3 mo post-FMT Δ fecal calprotectin μg/g	3 mo post-FMT Δ Mayo score	3 mo post-FMT Δ Endo score	3 mo post-FMT Δ UC Medications
**UC + CDI**	FMT2-1R	+90.7	-49.6	0	0	d/c steroids
	FMT11-1R	-45.2	-41.3	-4	0	changed to Entyvio
	FMT18-1R	+190.5	+82.9	N.A.	N.A.	+ anti-TNFα biologic
**UC**	FMT1-1R	-43.7	+47.5	0	-1	d/c steroids
	FMT3-1R	-23.8	N.A.	N.A.	N.A.	elective colectomy
	FMT7-1R	-46.6	-19.8	N.A.	N.A.	no change
	FMT8-1R	-14.1	-30.6	-2	-1	↓steroids
	FMT19-1R	-13.5	N.A.	N.A.	N.A.	no change

## Discussion

We report here our 16S rRNA sequence analysis of a pilot single donor fecal microbial transplant study of 19 recipients with recurrent CDI and/or UC. Because this pilot study was a prospective longitudinal observational study without a placebo control, it was not designed to address clinical efficacy. The analysis implemented in this pilot study considers all data points simultaneously in order to have the optimal degree of freedom for hypothesis testing. In addition we have adjusted for the multiple testing issue. Although we sequenced sufficiently deeply (mean > 100,000 16S rRNA sequences per sample) to identify all but the very rarest of OTUs, we were conservative in the number of OTUs included in the analysis. This was so that we could avoid statistical modeling issues because of excessive zero counts and control the false positive rate, given the small number of patients studied. Our analysis of this pilot study revealed marked alterations in the pre-FMT fecal microbiota composition in the CDI-only and CDI + UC recipients compared to their donors. These differences were characterized by depletion of anaerobic phyla/subphyla and correspondingly increased microaerophilic phyla/subphyla and a trend towards reduced community complexity (i.e., Shannon H). These findings are consistent with those reported by recent studies [26–30]. In contrast, the pre-FMT alterations in fecal microbiota in the UC-only recipients were less evident [31, 32].

One potential explanation for the marked dysbiosis observed in the CDI-only and CDI + UC recipients is that virtually all of these patients were on low dose intermittent oral vancomycin up to 48 h prior to the FMT (Table 1). However, observation of increased relative abundance of OTUs that are generally sensitive to vancomycin, such as *staphylococcus* and *streptococcus*, suggests that dysbiosis may not be simply attributable to vancomycin exposure. Instead, the marked imbalance between the relative abundances of microaerophilic and of strictly anaerobic taxa observed in the CDI-only and CDI + UC recipients may reflect increased intraluminal oxygen levels in the colon [33, 34].

The UC only and CDI + UC recipients were also on multiple medical regimens, which could confound the results of this analysis. The observed differences in β-diversity between UC only and UC + CDI however, are quite striking in this pilot study. These observations raise an important issue as to whether future studies on FMT in UC subjects should sub -phenotype their UC recipients with regard to whether there is a history of recurrent CDI. The observed differences in this pilot study may also explain why CDI was identified as a significant factor in our previous microbiome analysis of disease unaffected regions of resected ileum collected from ileal CD, colitis and non-IBD subjects [11].

Analyses conducted at a more granular level on individual genus-level OTUs identified several members of the *Enterobacteriaceae* family (Proteobacteria phylum) that exhibit increased relative abundances in all three groups (CDI-only, CDI + UC, and UC-only) of recipients. Increased abundances of members of the *Enterobacteriaceae* family have been associated with intestinal inflammation [35]. The question remains as to whether the increased load of *Enterobacteriaceae* is a result of intestinal inflammation or whether it plays a role in triggering inflammation, as has been observed in animal models [36, 37]. FMT reduces the relative abundance of these taxa in all three recipient groups.

The average relative abundance of the *Faecalibacterium* genus within the *Ruminococcaceae* (*Clostridia Group IV*) family, was markedly reduced in the CDI-only and CDI + UC recipients but not in the UC-only recipients. Its sole known species, *Faecalibacterium prausntizii* has a high relative abundance of ~5% and is involved in generating butyrate, which is an important nutrient for maintaining colonic mucosal homeostasis, and has anti-inflammatory properties. *F*. *prausntizii* has been reported to be depleted in patients with Crohn’s disease [38]. It remains to be determined whether preventing recurrent CDI-only after FMT can be linked to increasing the relative abundance of this specific taxa in prescreening potential donors for FMT and in developing these *F*. *prausnitzii* strains as probiotics for the treatment of recurrent CDI [39].

While this trial was not designed to address clinical efficacy, our findings reinforce previous reports that FMT is an effective treatment for preventing further recurrences of CDI. This may relate to the observation that the CDI-only and CDI + UC had marked dysbiosis prior to transplant. It is less clear, especially given the small number of recipients, whether FMT has a significant effect on the microbiological outcome of UC-only recipients where their baseline pre-FMT alterations in microbial structure/ composition compared to their donors were more subtle. The significant findings of this pilot study warrant a larger confirmatory study that carefully characterizes the microbial and molecular components of donor and recipient stools before and after FMT, with the goal of improving selection of recipients and donors for FMT, and to identify the active components of the transplanted stool for the development of new therapies for CDI and IBD.

## Supporting information

S1 Text Institutional Review Board (IRB)-approved trial study protocol. (PDF)

S1 Table Transparent Reporting of Evaluations with Nonrandomized Designs (TREND) checklist. (PDF)

## Abbreviations

FDA: US Food and Drug Administration
FMT: fecal microbiota transplant
IBD: inflammatory bowel diseases
mo.: month
nt: nucleotides
OTU: operational taxonomic unit
PCR: polymerase chain reaction
rRNA: ribosomal RNA
UC: ulcerative colitis
wk.: week
