# Supplementary material for: Longitudinal microbiome analysis of single donor fecal microbiota transplantation in patients with recurrent Clostridium difficile infection and/or ulcerative colitis
Source: PLoS One. 2018 Jan 31;13(1):e0190997. doi: 10.1371/journal.pone.0190997 (PMC5791968; doi:10.1371/journal.pone.0190997)
Supplement: S1 Text — (PDF) [file pone.0190997.s001.pdf]

S1 Text. Institutional Review Board (IRB)-approved trial study protocol.

TITLE: Fecal microbial transplantation in patients with medication refractory Clostridium difficile and/or Ulcerative colitis or indeterminate colitis

INVESTIGATORS:

Anupama Chawla M.D. (Principal Investigator)	Professor of Pediatric Gastroenterology
Ellen Li M.D.	Professor of Medicine/Gastroenterology
Jeffrey Morgansten M.D.	Assistant Professor of Pediatric Gastroenterology
Grace Gathungu M.D.	Assistant Professor of Pediatric Gastroenterology
Rupinder Gill, M.D.	Assistant Professor of Pediatric Gastroenterology
Sameer Lapsia, M.D.	Fellow in Pediatric Gastroenterology
Michelle Edelman, M.D.	Fellow in Pediatric Gastroenterology
Ramona Rajapakse, M.D.	Associate Professor of Medicine/Gastroenterology
Jonathan Buscaglia, M.D.	Assistant Professor of Medicine/Gastroenterology
Robert Richards, M.D.	Associate Professor of Medicine/Gastroenterology
Juan Carlos Bucobo, M.D.	Assistant Professor of Medicine/Gastroenterology
Paula Denoya, M.D.	Assistant Prof of Surgery/Colon Rectal Surgery
Jennifer Hsieh, M.D.	Fellow in Medicine/Gastroenterology
Maryann Parrish R.N.	Research Nurse Coordinator Gastroenterology
Donald Pettet, B.A.	Research Assistant Medicine/Gastroenterology
Leahana Rowehl	Research Assistant Medicine/Gastroenterology

A. SPECIFIC AIMS:

The following hypothesis will be tested in this study:

1. Fecal microbiota transplantation is a safe, tolerable, and efficacious procedure.
2. The fecal microbial composition and function in stool recipients after fecal transplantation will change to a similar composition and functionality as found in donor stool.

Primary objectives:

1. To determine the short term safety and tolerability of fecal microbiota transplantation in patients with recurrent or refractory Clostridium difficile and medication refractory Ulcerative colitis or indeterminate colitis up to 12 weeks post-transplant.

Secondary objectives:

1. To determine the long term safety and tolerability of fecal microbiota transplantation in patients with recurrent or refractory Clostridium difficile and medication refractory Ulcerative colitis or indeterminate colitis up to 1 year post-transplant.
2. To determine the efficacy of fecal microbiota transplantation in patients with recurrent or refractory Clostridium difficile and medication refractory Ulcerative colitis or indeterminate colitis in reducing or eliminating clinical symptoms.
3. To determine eradication of Clostridium difficile by negative PCR and/or toxin at 2 weeks post-transplant.
4. To determine change in fecal microbiome in patients treated with fecal microbiota transplantation.

B. BACKGROUND AND SIGNIFICANCE

Clostridium difficile is the leading cause of antibiotic-associated diarrhea with increasing infection rates and economic burden in developed countries. [1-4] According to the United States Healthcare Cost and Utilization

Project Kids' Inpatient Database (HCUP-KID), there has been an increase in pediatric *Clostridium difficile* from a rate of 7.24/10000 hospitalizations in 1997 to 12.80/10000 hospitalizations in 2006.[5] As the *C. difficile* epidemic worsens, the numbers of failed treatments and patients who experience relapses or recurrences also continues to grow. Patients being treated with usual first-line treatments such as metronidazole and vancomycin are encountering "hypervirulent" and resistant strains of *C. difficile* such as North American Pulsed Field type 1 (NAP1), restriction-endonuclease analysis (REA) type BI, or polymerase-chain reaction ribotype 027 (referred to collectively as the NAP1/B1/027 strain).[6] The NAP1/B1/027 strain of *C. difficile* has been deemed "hypervirulent" for its ability to produce binary toxin *C. difficile* 126 adenosine diphosphate-ribosyltransferase not typically found in other strains of *C. difficile*. This, in combination with its ability to produce excessive quantities of enteroxins A and B, compared with other strains of *C. difficile*, makes it hypervirulent[7] Even the newer agent, fidaxomicin, which was approved by the FDA in 2011, has a similar effectiveness to vancomycin with respect to the clinical resolution of acute diarrheal disease due to *C. difficile*.[6] Fidaxomicin, was however associated with a significantly lower rate of reoccurrence of *C. difficile* infection with non-North American Pulsed Field type 1 strains.[7] Fecal microbiota transplantation (FMT), the transfer of gastrointestinal microbiota from a healthy donor via infusion of a liquid stool suspension, is proving to be an effective alternative intervention to medication refractory *Clostridium difficile*. This may aid in decreasing the recurrent use of antibiotics, repeated hospitalizations, need for colectomy, and even death in patients with medication refractory *C. difficile*.[8]

Ulcerative colitis (UC) is a chronic, relapsing and remitting disease affecting the colon without a medical cure. The precise etiology of inflammatory bowel disease such as UC and Crohn's disease is unclear, however, its development, progression and phenotype are thought to be multifactorial with genetics and environment playing an important role.[9] UC is characterized by decreased prevalence of protective bacteria and concomitant increase in detrimental bacteria in the colon.[10] Evidence suggests that there is reduced diversity and richness of the colonic microbiome in UC with a decrease in *Firmicutes* such as bifidobacteria, lactobacillus, and *Faecalibacterium prausnitzii*.[11] *Firmicutes* are major producers of short-chain fatty acids such as butyrate, which is a substrate with immunoregulatory properties.[9] Inflammation in the colon in patients with IBD is hypothesized to result from an inappropriate activation of the mucosal innate immune system because of dysbiosis in genetically susceptible individuals.[10] A study by Frank et al evaluating the genotype of 178 patients (35 with Crohn's disease, 35 with Ulcerative Colitis, and 54 controls) found that IBD phenotype, NOD2 composite genotype and ATG16LI genotype were significantly associated with shifts in microbial compositions.[12] This study supports the concept that disease phenotype and genotype is associated with compositional changes in intestinal-associated microbiota .The treatment of IBD are medications such as corticosteroids, aminosalicylic acid preparations, immunomodulators, and biologics such as anti-tumor necrosis factor-α (TNF-α). However, each of these medications can have significant adverse side effects and many patients become refractory to these standard therapies and require surgery.[9] Given the role of the gastrointestinal microbiota in driving inflammation in patients with inflammatory bowel disease, treatments that manipulate the microbiota have been investigated including the use of probiotics such as VSL#3 and prebiotics.[9] However, not all probiotic formulations contain the same species of bacteria and their efficacy is variable. Recent evidence suggests that performing fecal microbiota transplantation (FMT) to restore the microflora of a diseased individual is efficacious in patients with medication refractory Inflammatory Bowel Disease.[11] Additionally, FMT has been used successfully in patients with irritable bowel syndrome, idiopathic constipation and a variety of non-GI diseases.[13] The first published case of FMT for UC was in January 1989 by Bennet and Brinkman.[14] Since then, there have been numerous case reports documenting its effectiveness in patients with UC.[15] A recent article by Kunde et al evaluating fecal transplantation in children and young adults with ulcerative colitis found the method to be well tolerated, feasible, and safe.[10]

C. PRELIMINARY STUDIES

Transplantation of stool for the treatment of various gastrointestinal disorders was first reported in 4th century China by Ge Hong, who described the use of human fecal suspension by mouth for patients who had food poisoning or severe diarrhea.[16] The first use of fecal enemas in humans for treatment of pseudomembranous colitis was reported in 1958 and for *Clostridium difficile* in 1983 by Schwan et al.[17] The first fecal

transplantation for treatment of *C. difficile* using colonoscopy was performed in 2010.[18] To date, more than 400 cases of fecal microbiota transplantation have been reported worldwide including approximately 75% by colonoscopy or retention enema and 25% by nasogastric or nasoenteric tube or by gastroduodenoscopy.[19]

A recent systematic review and meta-analysis conducted by Kassam et al evaluated eleven studies with a total of 273 patients with recurrent *Clostridium difficile* and found that clinical resolution occurred in approximately 90% of the patients undergoing fecal microbiota transplantation.[4] The authors found that that there were higher clinical resolution rates when the mode of delivery of the donor stool was via colonoscopy rather than nasoduodenal administration. Additionally, there were no reported adverse events associated with transplantation and follow-up was variable from weeks to years. A long-term (range 3-68 months) retrospective study of 77 patients who had undergone stool transplantation found that 91% of the patients maintained clinical resolution of symptoms 3 months post-transplant.[11] In this study, some patients did develop transient gastrointestinal symptoms or altered bowel habits for several days after fecal bacteriotherapy including the absence of bowel movements, and presence of abdominal cramping, gurgling bowel sounds, and increased feelings of gaseousness. Of the 77 subjects in this study, an autoimmune disease (rheumatoid arthritis, Sjögren syndrome, idiopathic thrombocytopenic purpura, and peripheral neuropathy) developed in 4 patients at some time after the transplant; however a clear relationship between the new disease and transplantation was not evident. Twenty immunosuppressed patients who were either taking glucocorticoids, immunosuppressive (6-mercaptopurine, azathioprine), or biologic (infliximab, adalimumab) agents or had diseases associated with immunocompromise also tolerated the transplantation well without any adverse events.[11] A recent article by Kunde et al evaluating fecal transplantation in 10 children and young adults with mild-to-moderate ulcerative colitis found the method to be well tolerated, feasible, and safe.[10] In these patients, mild (cramping, fullness, flatulence, bloating, diarrhea, and blood in stool) to moderate (fever) adverse events were self-limiting. The patients were followed for up to 4 weeks after FMT and 7 of the 9 stool recipients showed clinical response within 1 week and 6 of the 9 patients maintained clinical response at 1 month. 3 of the 9 patients achieved clinical remission at 1 week post-FMT.[2] According to a systematic review evaluating 17 articles reported on 41 patients with inflammatory bowel disease (27 with UC, 12 with Crohn's disease, and 2 unclassified) with a follow-up period between 2 weeks-13 years, FMT was effective and safe.[9]

The Division of Pediatric Gastroenterology at Stony Brook University Medical Center performed a fecal microbiota transplant in a 14 year old male with Ulcerative Colitis refractory to steroids and TNF-α antagonists. He responded well to the transplant with no adverse effects and improvement in his clinical symptoms for 3-4 weeks post-procedure. However his response was not sustained. FMT was also performed in a 12 year old female with *C. difficile* associated diarrhea and ulcerative colitis refractory to three courses of antibiotics (vancomycin, metronidazole, and fidaxomicin). She responded well to the transplant with no adverse effects and improvement in her clinical symptoms a few days after the procedure. Follow-up at 3 months shows no new *C. difficile* symptoms.

D. RESEARCH DESIGN AND METHODS

1. Rationale/overview:

The rationale for fecal microbiota transplantation in patients with refractory *Clostridium difficile* and/or Ulcerative Colitis or indeterminate colitis is its ability to reconstitute the normal microbial homeostasis leading to resolution or improvement of symptoms caused by the bacteria or underlying inflammatory bowel disease. This is becoming an especially important treatment modality as antimicrobial therapy for *Clostridium difficile* is becoming less efficacious due to resistant strains such as the hypervirulent NAP1/ribotype 027 strain.

Currently long-term follow-up data on patients who have undergone FMT is lacking. There are only scattered reports documenting the alteration of the enteric microbial composition and function status post transplantation. This study will significantly increase our understanding of the short and long term safety, efficacy, and tolerability of FMT. It will also enable us to assess the change in the fecal

microbiome after transplantation. Answers to these questions may lead to identification of key bacterial taxa to target future therapy.

2. Research Site:

Recruitment will occur at Stony Brook University Medical Center (SBUMC) in both the hospital and outpatient Pediatric and Adult Gastroenterology Clinic setting. The fecal microbiota transplantation will occur in the Endoscopy Suite on the 14th floor of SBUMC. Dr. Ellen Li's laboratory will be used to process stool and prepare it for transplantation. Her lab will also be utilized for collection, accession, and processing of patient based samples.

3. Study sample:

The study sample will consist of both pediatric and adult patients who meet eligibility criteria as outlined in the next section below on screening.

4. Screening:

SELECTION AND ENROLLMENT OF TRANSPLANT RECIPIENTS

Inclusion Criteria for Ulcerative colitis or Undifferentiated colitis:

All patients greater than 7 years of age with medication refractory Ulcerative Colitis or undifferentiated colitis who have failed standard medical therapy for at least one month

Inclusion Criteria for medication refractory *C. difficile*:

a) Patients greater than 7 years of age with recurrent or relapsing *C. difficile*:
   i. At least two episodes of mild-to-moderate *C. difficile* diagnosed via positive PCR or toxin and failure of standard treatment with at least two different antimicrobial agents used to treat *C. difficile* such as metronidazole, vancomycin, and/or fidaxomicin.
   ii. At least two episodes of severe *C. difficile* within 6 months resulting in hospitalization and associated with significant morbidity.
b) Patients greater than 7 years of age with unresponsive *C. difficile*:
   i. Moderate *C. difficile* colitis not responding to successive standard therapy (e.g. metronidazole, vancomycin, and/or fidaxomicin) lasting at least 28 days.
   ii. Severe and/or fulminant *C. difficile* colitis with no response to standard therapy after 48 hours.

Mild, moderate, and severe *C. difficile* colitis will be defined as <3, 3-6, and >6 diarrheal stools respectively per day

All patients or legal guardians of patients less than 18 years of age will have to give consent. The consent form will outline that although fecal microbiota transplantation appears safe based on past studies, a theoretical risk of transmission of an unrecognized infectious agent or substance exists and could result in an unexpected disease.

Exclusion Criteria:

a) Patients less than 1 year of age
b) Scheduled for abdominal surgery within the next 12 weeks
c) Pregnancy
d) Grade 4 anemia (Hemoglobin < 6 g/dL)[20]
e) Grade 1 neutropenia (Absolute Neutrophil Count <1500)[20]
f) Known diagnosis of Graft vs. host disease
g) Major abdominal surgery within the past 3 months
h) Administration of any investigational drug within the past 2 months
i) Use of a TNF-α antagonist within 2 weeks of the proposed date of transplantation

SELECTION AND ENROLLMENT OF TRANSPLANT DONORS

Inclusion Criteria:
Spouses, parents, family members, friends, or associates of the stool recipient who consent to the screening tests outlined below:

a) Stool testing:
   i. *Clostridium difficile* toxin B by PCR; or evaluation for toxins A and B by EIA
   ii. Routine bacterial culture for enteric pathogens
   iii. Fecal *Giardia* and *Cryptosporidium* antigens
   iv. Acid-fast stain for *Cyclospora, Isospora*
   v. Ova and parasites
   vi. Helicobacter pylori fecal antigen (for upper gastrointestinal routes of fecal microbiota transplantation)

b) Serologic testing:
   i. HIV, type 1 and 2
   ii. HAV IgM
   iii. HBsAg, anti-HBc (both IgG and IgM), and anti-HBs
   iv. HCV Ab
   v. RPR and FTA-ABS

Exclusion Criteria:
Individuals with:
a) Known HIV, Hepatitis B, or C infections
b) Known exposure to HIV or viral hepatitis (within the previous 12 months)
c) High-risk sexual behaviors (examples: sexual contact with anyone with HIV/AIDS or hepatitis, sex for drugs or money)
d) Known use of illicit drugs within the past 3 months
e) Tattoo or body piercing within the past 6 months
f) Incarceration or history of incarceration
g) Known febrile illness within the past 2 weeks of the proposed date of stool donation or current communicable disease (example: upper respiratory tract infection)
h) Risk factors for variant Creutzfeldt-Jakob disease
i) Travel (within the last 3 months) to developing countries
j) History of inflammatory bowel disease or chronic diarrhea (i.e. greater than 3 loose stools daily for the past 3 months)
k) History of gastrointestinal malignancy or known polyposis
l) Systemic antibiotics within the preceding 3 months
m) Current use of major immunosuppressive medications (e.g., calcineurin inhibitors, systemic anti-neoplastic, exogenous glucocorticoids, biologic agents)
n) Recent ingestion of a potential allergen (e.g., nuts) where recipient has a known allergy to this (these) agent(s) within one week prior to the fecal transplant
o) Any autoimmune disease, moderate to severe malnutrition (BMI<15.0), chronic pain syndrome, metabolic syndrome, or a neurologic or neurodevelopmental disorder
p) Atopic disease requiring steroids or immune modulating therapy
q) Found to have any of the conditions tested below for pre-screening purposes

Donor testing:
All donors will be screened and tested for relevant communicable diseases that may be passed to the transplant recipient. A communicable disease is deemed relevant if 1) it is one for which there may be a

risk of transmission by stool either to the patient or to those people who may handle or otherwise come in contact with the stool; and 2) it could be fatal or life-threatening, could result in permanent impairment of a body function or permanent damage to body structure, or could necessitate medical or surgical intervention to preclude permanent impairment of body function or permanent damage to a body structure; and 3) it is one for which appropriate screening measures have been developed and/or an appropriate screening test for donor specimens have been licensed, approved, or cleared for such use by FDA and is available.

c) Stool testing:
  i. *Clostridium difficile* toxin B by PCR; or evaluation for toxins A and B by EIA
  ii. Routine bacterial culture for enteric pathogens
  iii. Fecal *Giardia* and *Cryptosporidium* antigens
  iv. Acid-fast stain for *Cyclospora, Isospora*
  v. Ova and parasites

d) Serologic testing:
  i. HIV, type 1 and 2
  ii. HAV IgM
  iii. HBsAg, anti-HBc (both IgG and IgM), and anti-HBs
  iv. HCV Ab
  v. RPR and FTA-ABS

5. Procedure Protocol:

Initial Recruitment: We will collect baseline clinical data at the initial screening visit (either in the inpatient hospital setting or outpatient Adult or Pediatric Gastroenterology clinic) to determine eligibility (Appendix A1/A2). This will include past medical history in terms of previous episodes of *C. difficile* associated disease and inflammatory bowel diseases (age of diagnosis, duration, medications) and standard clinical information such as age, sex, race, disease phenotype, medications, and smoking history. After the patient is deemed eligible for the study, he/she or the guardians of the patient will be asked for potential donors. The donors will be screened according to the criteria listed above. A checklist will be used to assess for pre-screening eligibility of the donor (Appendix B). Once the checklist is complete, the donor is deemed eligible if he/she answers **no** to each question. He/she will then be screened via stool and serological testing to assess for infectious pathogens that may theoretically be passed from the donor to the recipient in the stool. This testing will be covered by the donor's insurance. This will be performed within 2 weeks of the proposed FMT. Only if the results of the stool and blood tests are negative showing no infectious pathogens, will the donor be able to donate stool to the recipient. Date of the FMT will be established.

Week before FMT: The recipients and donors will be asked to record their diet using a diary provided by the investigators, during the week that they provide stool samples (Appendix C). The purpose of this is to ensure that the donor does not ingest any foods that may be potentially allergenic to the recipient. The ingested foods may also impact the microbial load of the donor stool. (*Please note stool, serum, and/or saliva collection for research purposes are optional and not required for participation in the study. Those patients that do not consent will only be analyzed on safety, tolerability, and efficacy of FMT and not whether the fecal microbiome has been altered).*

Blood, saliva, and stool handling for research purposes is discussed in section below titled: Patient Sample Collection and Processing

Pre-transplant stool collection: 1-3 days before FMT, the recipient's stool will be collected. This will be performed using stool kits as described in Human Microbiome Project – Core Microbiome Sampling Protocol A (http://www.ncbi.nlm.nih.gov/projects/gap/cgi-bin/GetPdf.cgi?id=phd002854.2), and used within 6 hours of passage. By using the stool kit, the patient will place an aliquot of stool in a stool specimen container and another aliquot in a stool specimen container filled with 10 ml RNAlater (Qiagen), an RNA stabilization solution, to better improve recovery of bacterial and human nucleic acids during the transport period. The kits given to the recipient will include ice packs to keep the stool refrigerated until he/she reaches the hospital to be processed. Upon receipt of the stool, the stool will be processed in a biologically safety hood located within GI Research Space on the 17th floor of the Health Sciences Center Building at Stony Brook using universal precautions and given a coded identifier. *Please note stool, serum, and/or saliva collection for research purposes are optional and not required for participation in the study. Those patients that do not consent will only be analyzed on safety, tolerability, and efficacy of FMT and not whether the fecal microbiome has changed.*

Day before transplant:
The stool recipient will be given instructions for a bowel prep based on the patient's weight. Participants and/or guardians will be told that in order to properly visualize their intestines, they must be "prepped" or flushed of their contents. These medications will cause diarrhea which is the desired effect. The recipient should be in an area with easy access to the bathroom when the medication is given. The goal is to be passing clear fluid without any formed stool. The recipients will be told to call the study coordinator if this does not occur, so that it can be determined whether any additional medications are necessary for preparation**. Once the prep has begun, the recipient will be told to be on a diet consisting of clear liquids only. Clear liquids are those that they can see through and they will be told to avoid red, orange or purple liquids.** Acceptable liquids include water, ginger ale, chicken broth (no chicken or noodles), apple juice, Pedialyte, or jello. **For the last 7 hours prior to the procedure, the recipient should have nothing to eat or drink.** Medications may be taken with a very small amount of water.

The colon cleanout prep will be determined by the patient's gastroenterologist. The recipient should be passing clear stools by 5 PM day before FMT. If the recipient is not passing loose to watery clear stools by this time, they will be told to call the study coordinator or principal investigator.

Day of transplant: Stool collection/handling (adapted from Bakken et al)[8]:

- The donor must have been afebrile (temperature <100.4°C) in the preceding 2 weeks and must not have ingested a potential recipient allergen for the FMT to proceed.
- The donor will be told to collect stool using Saran™ Wrap, plastic hat, or a collection container. The stool will need to be collected prior to any contamination with the toilet bowl.
- The stool will then be kept in an airtight container
- The stool may be kept at room temperature if used within 6 hours or kept at 4 degrees Celsius to be used up to 24 hours. Stool sample must not be frozen

Stool processing:

- Once the stool arrives at Stony Brook University Medical Center, a Case Report form (Appendix D) describing the stool consistency (hard vs. soft) and stool appearance (bloody vs. nonbloody, presence/absence of mucous) will be logged in by a laboratory technician and assigned a label
- The stool will then be transported to a biological safety hood located within GI Research Space on the 17th floor of the Health Sciences Center Building at Stony Brook to process the stool

- The majority of the stool will be used for FMT and 2 aliquots of the stool will be used to perform microbiome studies (*again the microbiome portion of this study is optional and will not preclude FMT*)

Stool preparation:

- Stool preparation will occur in the biological safety hood
- Universal precautions will be adhered to. Those involved with mixing and/or handling the fecal transfusion material will wear a fluid-resistant gown, gloves, and mask with goggles, or eye shield
- Donor stool will be transferred to a single-use disposable container
- Preservative-free normal saline (250-500 mL) at room temperature will be used to dilute the stool sample until it reaches a liquid slurry consistency
- The stool slurry will then be filtered to remove as much particulate matter as possible. This will be accomplished using gauze pads lining the inside portion of a plastic disposable funnel
- The filtered stool slurry will be divided into aliquots of 50 mL and will be used within an hour of preparation

Stool instillation via Endoscopy for Clostridium difficile and/or Ulcerative colitis or undifferentiated colitis:

- The volume to be instilled will be 250-500 mL from the terminal ileum/cecum to the rectum. Currently, Stony Brook University Medical Center will administer the stool via colonoscopy but in the future may also administer smaller volumes (e.g., 25-50 mL) via nasoduodenal-tube into the duodenum of the recipient.
- After the transplant, the patient will be observed in the Endoscopy Suite for 1-2 hours via continuous monitoring.

Stool instillation via Enema(s) (adapted from Kunde et al)[10]:

- Recipients who elect to have FMT via retention enemas will receive the donated stool over 1 hour (60 mL enema will be given every 15 minutes, with each enema given infused over 5 minutes)
- The recipient will be placed in the left lateral decubitus position with elevated hips in a designated private room in the Endoscopy Unit at Stony Brook University Medical Center when the enema is administered
- After the transplant, the patient will be observed in the Endoscopy Suite for 1-2 hours via continuous monitoring

For research purposes: 20 mL of whole blood and/or 1-2 mL of saliva will be collected from the recipient. *(Please note stool, serum, and/or saliva collection for research purposes are optional and not required for participation in the study. Those patients that do not consent will only be analyzed on safety, tolerability, and efficacy of FMT and not whether the fecal microbiome has changed).*

Follow-up:
Safety, efficacy, and tolerability will be recorded on a routine basis. Checklists will be incorporated assessing general well-being of the patients and adverse events during weekly follow-up telephone calls as well as clinic visits (Appendix E-F). These checklists will be completed via telephone call the day after the procedure, weekly thereafter for 12 weeks and then monthly until 1 year post-transplant.

Additionally clinic visits will be conducted at 1, 3, 6, 9, and 12 months post-transplant for in-depth evaluation of the patient. Any serious adverse events will be documented (Appendix G). Additionally, during clinic visits, the Pediatric Ulcerative Colitis Disease Activity Index (PUCDAI) (Appendix H) and the Mayo Scores (Appendix I) for measuring disease activity in the subjects will be used as a clinical outcome.

These checklists will contain a prompt to inform the study principal investigator of any adverse events. Patients will be triaged to either the Emergency Department or to Adult or Pediatric Gastroenterology Clinic if any concerning adverse events occur. Any Serious Adverse Event will be evaluated by the Principal Investigator and brought to the attention of the Data Safety Monitoring Board.

Recipient stool collection for microbiome portion of study will occur 1 week after FMT and at the 3 month clinic visit if the recipient has consented to this portion of the study.

Patient Sample Collection and Processsing: We will collect blood, saliva and stool samples using the same protocols we have put in place for collection of these samples for the Stony Brook University Digestive Diseases Research Tissue Procurement Facility (IRB net ID: 163184-15). If the patients undergoing endoscopy will also consent to research endoscopic biopsies we will collect these biopsy samples using a separate consent for contribution to the Stony Brook University Digestive Diseases Research Tissue Procurement Facility (IRB net ID: 163184-15). The fecal, saliva and blood samples and downstream products (e.g. DNA and RNA) that are not utilized by the present study will be stored until the samples are exhausted within the Stony Brook University Digestive Diseases Research Tissue Procurement Facility (IRB net ID: 163184-15). The clinical data, stripped of patient health identifiers, will be assigned a patient ID and a visit ID and will be stored in the Stony Brook University Digestive Diseases Research Tissue Procurement Facility clinical database. The patient samples will also be stripped of patient health identifiers and assigned a patient ID, visit ID and sample ID and will be archived within the Stony Brook University Digestive Diseases Research Tissue Procurement Facility.

1. **Genotyping for IBD risk alleles.** DNA will be extracted from peripheral mononuclear blood cells (in blood) or saliva as previously described.[21] The DNA will be assigned the same patient ID, visit ID and sample ID from the original sample. We have used Taqman PCR assays, Sequenom, and array methods for analyzing genotypes thus far, but the technology is rapidly evolving.[22] We will seek the most cost effective means of obtaining accurate genotyping of established IBD risk alleles. If we submit the samples for genotyping at another facility, each of these samples will be assigned another sample ID, to further protect the privacy of the subjects, prior to shipping these samples to an outside facility.
2. **16S rRNA sequencing.** 16S rRNA sequencing will provide a first tier of microbiome surveillance.[23] This may involve shipping the stool and possible tissue DNA to outside facilities for library construction (University of Colorado) and sequencing (Cold Spring Harbor Laboratories). If we submit these DNA samples to outside institutions for analysis, each of these samples will be assigned another sample ID, to further protect the privacy of the subjects, prior to submission of these samples to an outside facility. We propose to measure the alterations in microbial composition before and after fecal microbial transplantation.
3. **Targeted qPCR sequencing.** To detect targeted microbial groups such as *C. difficile*, we will utilize targeted qPCR using the same kits for detecting *C. difficile* toxin B that are employed in molecular pathology at Stony Brook University Medical Center.[24]
4. **Fecal calprotectin.** Fecal calprotectin is a marker of intestinal inflammation that will complement the colonoscopic scores and patient questionnaires. We propose to measure fecal calprotectin in each of the collected stools using the PhiCal commercial ELISA kit as previously described.[25] Frozen stools archived at -80°C will be batch extracted and analyzed along with the manufacturer's control to avoid interassay variability.

5. **Shotgun metagenomics, bacterial metatranscriptomics, bacterial metaproteomics.**[22, 26] We propose to conduct multi-omic studies on microbial function using the collected stool samples if we are successful in obtaining outside funding to support these studies. This may require sending aliquots of samples or their downstream products to other institutions for analysis. If so, the samples will be assigned another sample ID to further protect the privacy of the subjects before shipping these samples out to other institutions.

Flowsheet for the Full Study Protocol is outlined in Appendix J.

E. STATISTICS:

This is an exploratory pilot study to generate preliminary 'omics data for the NIH Microbiome Grants so that a power analysis can be conducted. It is anticipated that 10 patient data is needed in order to generate sufficient preliminary data to conduct a power analysis. Number of patients enrolled will not be limited as the primary goal of this study is to establish safety and tolerability of FMT.

F. FUNDING STATUS, DETAILS:

Simons Foundation to Dr. Ellen Li. We are seeking National Institutes of Health (NIH) funding from U54 submission February 8, which will incorporate funding for this study.

G. HUMAN SUBJECTS RESEARCH PROTECTION FROM RISK

Risk to Subjects:

The risks associated with the colonoscopy are infection, bleeding, and less than 1% chance of perforation. Additionally, there are risks associated with receiving anesthesia during colonoscopy including anaphylaxis and respiratory distress. Collection of stool, blood, and/or saliva is minimally invasive. The risk of collecting blood is infection at the site of needle stick and bruising. As per Kunde et al who performed a study on fecal microbiota transplantation in 10 children with mild-to-moderate Ulcerative colitis, no serious adverse events occurred in the patients.[10] The minimal symptoms that occurred bloating/flatulence, abdominal pain/cramping, diarrhea, blood in the stool, fatigue, and fever.'

Risk of collecting data: Extremely rare possibility that the patient data can be shared in a way that identifies the patient. Great lengths will be made to restrict access to the patient code. If the patients undergoing colonoscopy for clinical indications, are willing to donate research endoscopic biopsies, they will be consented separately for research endoscopic biopsies for contribution to the Stony brook University Digestive Diseases Research Tissue Procurement Facility. Please note that obtaining consent for research biopsies is optional and is not required for participation in this study.

Adequacy of Protection Against Risks:

The clinical data and stool samples will be stripped of patient health identifiers and assigned a patient code and sample code. The subject's participation will end after the collection of stool and clinical data is completed 1 year after enrollment in the study. We are only requesting that we be able to collect clinical metadata by prospective questionnaires and reviewing the medical records, and to collect fecal, saliva and blood samples before and after therapy. The consents will be obtained by the physicians or our nurse coordinator, who have been trained in good clinical practices and are highly experienced in providing and obtaining informed consent in this patient population.

Additionally, all patients will have adverse event monitoring:

Day of fecal microbiota transplantation:

a) During the procedure:
   i. If endoscopy or colonoscopy is performed, an anesthesiologist will be assigned to the recipient and the endoscopy unit will have full-code preparedness

ii. If enemas are given, the recipient's comfort and willingness to proceed with the next enema will be assessed after each enema infusion

b) Postoperative:
   i. If endoscopy or colonoscopy is performed, the recipient will be monitored in the post-operative area of the endoscopy suite for one hour by continuous monitoring
   ii. If the patient received FMT via enema, the patient will be monitored for one hour post enema instillation

Short term monitoring (First 3 months):

a) 1 day post-transplant: The transplant recipient will receive a phone call the day after the procedure and questions will be asked from a standardized checklist containing the following items:
   i. General well being
   ii. Presence of abdominal pain
   iii. Presence of temperature greater than 100.4 degrees Celsius
   iv. Presence of diarrhea (loose, watery stools) or blood in the stool
   v. Presence of new rash

A logarithm will be attached to the checklist to refer patients to either clinic or the Emergency Department

b) Weekly monitoring: All recipients will have weekly monitoring via telephone call for 12 weeks. This will include the following additional questions:
   i. Has diarrhea stopped or improved?
   ii. If the diarrhea had stopped, is there a recurrence of diarrhea?
   iii. If the diarrhea has not improved, have you tried other treatments?
   iv. Has there been any need of antibiotics since the transplant?
   v. Did any medical condition you had before your fecal transplant go away after your fecal transplant? (for example, arthritis and chronic skin rash)
   vi. Has abdominal pain if present prior to fecal microbiota transplant resolved?
   vii. Have you developed any new medical conditions since the fecal transplant?

c) A follow-up visit in-person study will be scheduled at 1 and 3 months post-transplant

d) Each patient will receive a diary card (Appendix K) where he/she or the patient's guardian will note when the stool recipient experiences a new symptom or exacerbation of current symptoms. This diary card will be reviewed when the patient is followed up in in clinic.

e) Patients will be advised to call either the Pediatric Gastroenterology or Adult Gastroenterology offices for any questions or health related concerns.

Long Term Monitoring (First year post-transplant):

a) After the first 12 weeks, all recipients will have monthly telephone calls up to 1 year post-transplant with the standardized checklist
b) A follow-up visit will be scheduled at 6 months, 9 months, and 1 year post-transplant
c) The diary card (Appendix K) given to the patient earlier will be reviewed at each clinic visit
d) Patients will be advised to call either the Pediatric Gastroenterology or Adult Gastroenterology offices for any questions or health related concerns for up to 1 year post-transplant

Potential Benefits of Proposed Research to the Subjects and Others:

The potential benefits of having the fecal transplantation for treatment of *Clostridium difficile* is improvement in the patient's diarrhea, abdominal pain, and eradication of the bacteria. The potential benefits of having fecal microbiota transplantation for ulcerative colitis or indeterminate colitis is improvement in manifestations of having inflammatory bowel disease such as abdominal pain, diarrhea, fatigue, rashes, joint pains, and/or appetite.

If the NIH grant is funded, the subjects and families will be compensated for their time in completing the questionnaires and submitting the stool and saliva samples. Conducting research on these samples, including the research proposed in this application may create new tests, treatments, or cures. If it does the patients who have donated samples will not receive any money from those products.

Importance of the Knowledge to be Gained:

This study will enable us to contribute to the literature regarding the safety, efficacy, and tolerability of fecal microbiota transplantation. Additionally, by collecting well phenotyped linked samples, future integrations of the microbiome data not only with phenotype but also with genetic data and metabolomics data will be possible. This will enable further studies investigating the mechanism(s) by which gut bacteria can affect susceptibility to medication refractory *Clostridium difficile* and/or ulcerative colitis or indeterminate colitis.

H. DATA SAFETY MONITORING PLAN (for more than minimal risk studies):

A data safety monitoring committee has been established and is composed of the three following experts:

a) Sharon Nachman, M.D., Chair of Data Safety Monitoring Board, Professor of Pediatrics, Chief, Division of Infectious Diseases, Stony Brook University School of Medicine. Expertise in clinical trials.
b) Roberto Bergamaschi, M.D., Professor of Surgery, Chief, Division of Colon Rectal Surgery, Stony Brook University School of Medicine. Expertise in Colon Rectal Surgery and consequently surgical treatment of colonoscopic complications.
c) Matthew Ciorba, M.D., Assistant Professor of Medicine, Division of Gastroenterology and Hepatology, Washington University School of Medicine. Expertise in probiotics in inflammatory bowel diseases.

The committee will be asked to perform an interim assessment of safety after four subjects are enrolled in the study. The committee will then perform a second safety assessment after another four patients (total 8) are recruited. This will help in determining whether or not this study protocol should continue to be implemented or requires modifications.

The following rules will govern the study team during review of recipient follow-up data. At any time one of the listed events occur, this will trigger the team to request a safety monitoring committee review of the data and pause further enrollment:

i. Death in any subject that is judged by the principal investigator as possibly, probably, or definitely related to fecal microbiota transplantation
ii. A life-threatening adverse event in any subject that is judged by the principal investigator as possibly, probably, or definitely related to fecal microbiota transplantation
iii. A serious adverse event possibly, probably, or definitely related to fecal microbiota transplantation as judged by the principal investigator
iv. A pattern of significant symptoms or physical symptoms that, although individually minor, collectively represent a safety concern in the opinion of the study team

An adverse event will be defined as any untoward medical occurrence associated with FMT, whether or not related to FMT. A serious adverse event will be defined as an adverse event that results in death, is life-threatening, or requires hospitalization. Adverse event will be recorded when follow-up phone calls are made and during study clinic visits. Intensity and relationship of adverse events with FMT will be described using Common Terminology Criteria for Adverse Events (CTCAE version 3.0) and Toxicity Grading Guidance from Vaccine Clinical Trials (U.S. Food and Drug Administration, September 2008). The intensity of adverse events will be classified as mild, moderate, and severe.

The data safety monitoring committee may recommend resumption of fecal microbiota transplantation if the study pause was for reasons less severe than those in the protocol-specified pausing rules listed above. The committee may also require changes to the protocol to ameliorate the safety concerns that pose significant risk to stool recipients. In the absence of protocol changes, the data safety monitoring committee must follow the protocol-specified stopping rules.

I. LITERATURE CITED:

1. Hopkins MJ, MacFarlane GT. Changes in predominant bacterial populations in human faeces with age and with Clostridium difficile infection. *J Med Microbiol* 2001; 51:448-454.
2. Chang JY, Antonopoulos DA, Kalra A, et al. Decreased diversity of the fecal microbiome in recurrent Clostridium difficile-associated diarrhea. *J Infect Dis* 2008; 197:435-438.
3. Bouza E. Consequences of Clostridium difficile infection: understanding the healthcare burden. *Clin Microbiol Infect*. *Suppl* 2012; 6:5-12.
4. Kassam Z, Lee CH, Yuan Y, et al. Fecal microbiota transplantation for Clostridium difficile infection: systematic review and meta-analysis. *Am J Gastroenterol* 2013; 108:500-508.
5. Pant C, Despande A, Altaf M, et al. Clostridium difficile infection in children: a comprehensive review. *Curr Med Res Opin*. Posted online on 9 May 2013.
6. Louie TJ, Miller MA, Mullane KM, et al. Fidaxomicin versus vancomycin for Clostridium difficile infection. *N Eng J Med* 2011; 364:422-431.
7. Lancaster JW, Matthews SJ. Fidaxomicin: The newest addition to the armamentarium against Clostridium difficile infections. *Clin Ther* 2012; 34:1-13.
8. Bakken JS, Borody T, Brandt LJ. Treating clostridium difficile infection with fecal microbiota transplantation. *Clin Gastroenterol Hepatol* 2011; 9:1044-1049.
9. Anderson JL, Edney RJ, Whelan K. Systematic review: faecal microbiota transplantation in the management of inflammatory bowel disease. *Aliment Pharmacol Ther* 2012; 36:503-516.
10. Kunde S, Pham A, Bonczyk S, et al. Safety, tolerability, and clinical response after fecal transplantation in children and young adults with ulcerative colitis. *J Pediatr Gastroenterol Nutr* 2013; 56:597-601.
11. Sartor RB. Microbial influences in inflammatory bowel diseases. *Gastroenterology* 2008; 134:577-594.
12. Frank DN, Robertson CE, Hamm CM, et al. Disease phenotype and genotype are associated with shifts in intestinal-associated microbiota in inflammatory bowel diseases. *Inflamm Bowel Dis* 2011;17:179-184.
13. Arionadis OC & Brandt LJ. Fecal microbiota transplantation: past, present and future. *Curr Opin Gastroenterol* 2013; 29:79-84.
14. Bennet JD & Brinkman M. Treatment of ulcerative colitis by implantation of normal colonic flora. *Lancet* 1989; 1:164.
15. Damman CJ, Miller SI, Surawicz CM, et al. The microbiome and inflammatory bowel disease: is there a therapeutic role for fecal microbiota transplantation? *Am J Gastroenterol* 2012; 107:1452-1459.
16. Brandt LJ, Aroniadis O. (2013) An overview of fecal microbiota transplantation: techniques, indications, and outcomes. *Gastrointest Endosc*. Article in press, 1-10.
17. Schwan A, Sjolin S, Trottestam U, et al. Relapsing Clostridium difficile enterocolitis cured by rectal infusion of homologous faeces. *Lancet* 1983; 2:845.
18. Brandt LJ, Aroniadis OC, Mellow M, et al. Long-term follow-up of colonoscopic fecal microbiota transplant for recurrent Clostridium difficile infection. *Am J Gastroenterol* 2012; 107:1079-1087.
19. Cohen SH, Gerding DN, Johnson S, et al. Clinical practice guidelines for Clostridium difficile infection in adults; 2010 update by the society for healthcare epidemiology of America (SHEA) and the infectious diseases society of America (IDSA). *Infect Control Hosp Epidemiol* 2010; 31:431-455.
20. Division of AIDS Table for Grading the Severity of Adult and Pediatric Adverse Events, Version 1.0, December 2004. 2004 [November 19, 2010]. http://rsc.tech-res.com/Document/safetyandpharmacovigilance/Table_for_Grading_Severity_of_Adult_Pediatric_Adverse_Events.pdf
21. Ananthakrishnan AN, Issa M, Binion DG. Clostridium difficile and inflammatory bowel disease. *Med Clin North Am* 2011; 94:135-53.
22. Giannoukos G, Ciulla DM, Huang K, et al. Efficient and robust RNA-seq process for cultured bacteria and complex community transcriptomes. *Genome Biol.* 2012; 13:R23.
23. Shahinas D, Silverman M, Sittler T, et al. Toward an understanding of changes in diversity associated with fecal microbiome transplantation based on 16S rRNA gene deep sequencing. *MBio.* 2012; pii: e00338-12.
24. Stamper PD, Alcabasa R, Aird D, et al. Comparison of a commercial real-time PCR assay for tcdB detection to a cell culture cytotoxicity assay and toxigenic culture for direct detection of toxin-producing *Clostridium difficile* in clinical samples. *J Clin Microbiol* 2009; 47:373–378.
25. Henderson P, Casey A, Lawrence SJ, et al. The diagnostic accuracy of fecal calprotectin during the investigation of suspected pediatric inflammatory bowel disease. *Am J Gastroenterol* 2012; 107:941-949.

26. Erickson AR, Cantarel BL, Lamendella R, et al. Integrated metagenomics/metaproteomics reveals human host-microbiota signatures of Crohn's disease. *PLoS One* 2012; 7:e49138.

APPENDIX A1 (Ulcerative Colitis or Indeterminate Colitis):

STUDY PATIENT CHARACTERISTICS

Within 2 weeks prior to Fecal Transplantation

PATIENT NAME: ____________________________ SEX: ☐M/☐F
DOB: ___ ___/___ ___/___ ___
Assigned Code Number: ____
WEIGHT: ____ KGS BMI: ____
HEIGHT: ____ CMS

**1.** Duration of symptoms related to Ulcerative colitis or indeterminate colitis	____ Month(s)		
**2.** Date of IBD diagnosis	__ __/__ __/__ __		
**3.** Current medications	Name		
**4.** Medications for IBD the patient has been treated with:	Name		Duration (days)
**5.** Probiotics the patient has been treated with:	Name		Duration (days)
**6.** Diarrhea (BMs/day):	<3	3-6	>6
**7.** Blood in stool:	Yes		No
**8.** Mucous in stool:	Yes		No
**9.** Abdominal pain (Scale 0-10)	___/10		
**10.** Fatigue	Yes		No
**11.** Weight loss	Yes ____KGs		No
**12.** Previous abdominal surgery	Yes Location___________		No
**13.** Smoker?	Yes Packs/day:__________		No

APPENDIX A2 (*Clostridium difficile*):

STUDY PATIENT CHARACTERISTICS

Within 2 weeks prior to Fecal transplantation

PATIENT NAME: ____________________________ SEX: ☐M/☐F
DOB: ___ ___/___ ___/___ ___
Assigned Code Number: ____
WEIGHT: ____ KGS BMI: ____
HEIGHT: ____ CMS

**1.** Duration of symptoms related to *C. difficile*	____ Month(s)		
**2.** *C. difficile* PCR and/or toxin date(s)	__ __/__ __/__ __<br>__ __/__ __/__ __		
**3.** Antibiotics for *C. difficile* the patient has been treated with:	Name		Duration (days)
**4.** Probiotics for *C. difficile* the patient has been treated with:	Name		Duration (days)
**5.** Diarrhea (BMs/day):	<3	3-6	>6
**6.** Blood in stool:	Yes	No	
**7.** Mucous in stool:	Yes	No	
**8.** Abdominal pain (Scale 0-10)	___/10		
**9.** Fatigue	Yes	No	
**10.** Weight loss during illness	___KGS		
**11.** Previous abdominal surgery	Yes<br>Location:___________	No	
**12.** Smoker?	Yes<br>Packs/day:__________	No	

APPENDIX B
Stool Donor Screening Questionnaire

Question	Yes	No
**1**. Do you have known HIV, Hepatitis B, or C infections?		
**2**. Have you been exposed to HIV or viral hepatitis (within the previous 12 months)?		
**3**. Do you engage in any high-risk sexual behaviors (examples: sexual contact with anyone with HIV/AIDS or hepatitis, sex for drugs or money)?		
**4**. Have you used illicit drugs within the past 3 months?		
**5**. Have you had a tattoo or body piercing within the past 6 months?		
**6**. Have you ever been incarcerated?		
**7**. Have you been to an area with Mad Cow Disease (risk factor for Creutzfeld-Jakob disease)?		
**8**. Have you traveled (within the last 3 months) to developing countries?		
**9**. Do you have a history of inflammatory bowel disease or chronic diarrhea (i.e. greater than 3 loose stools daily for the past 3 months)?		
**10**. Do you have a history of gastrointestinal malignancy or known polyposis?		
**11**. Have you needed to use systemic antibiotics within the preceding 3 months?		
**12**. Are you currently using any major immunosuppressive medications (e.g., calcineurin inhibitors, systemic anti-neoplastic, exogenous glucocorticoids, biologic agents)?		

APPENDIX C

Stony Brook University Medical Center Food Diary

Coded Number: _______________

Physician: _____________________

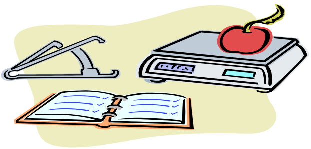

Record all food, fluid, supplement intake including Probiotics
Record all condiments, sauces, oils, etc.

Date:

MEAL	FOOD: Brand/Description (i.e. Whole milk, rye bread)	HOW WAS IT PREPARED (i.e. 3 ounces fried chicken)

****Record each day on separate sheet****

APPENDIX D:

DAY OF TRANSPLANT: CASE REPORT FORM

DATE OF STUDY: ___ ___/___ ___/___ ___ TIME OF STUDY: ___:___

DONOR CODED No.: ___________________________________

RECIPIENT CODED No.: ________________________________

DONOR DATA:

**1.** How do you feel today?	WELL	UNWELL
**2.** Have you had a fever > 100.4 degrees Celsius in the past 2 weeks?	YES	NO
**3.** Have you had cough or runny nose within the last 2 weeks?	YES	NO
**4.** Have you ingested ________ (recipient allergen) in the past week?	YES	NO

DONOR STOOL DATA:

**1.** What date and time was the stool produced?	__ __/__ __/__ __ AM/PM		
**2.** If produced > 6 hours before time of colonoscopy, was the stool kept at 4 degrees Celsius?	YES	NO	N/A
**3.** Was the stool collected prior to contamination with the toilet bowl?	YES	NO	
**4.** Is there presence of blood in the stool?	YES	NO	
**5.** Is there presence of mucous in the stool?	YES	NO	
**6.** What is the consistency of stool?	Hard	Soft	Liquidy

APPENDIX E:

DAY AFTER TRANSPLANT: CASE REPORT FORM

RECIPIENT CODED No.: ___________________
DATE/TIME OF CALL: ___ ___/___ ___/___ ___ ___:___AM/PM

RECIPIENT DATA:

**1.** How do you feel today?	WELL		**UNWELL**
**2.** Have you had a fever > 100.4 degrees Celsius in the past 2 weeks?	**YES**		NO
**3.** Is your abdominal pain:	BETTER	**WORSE**	SAME
**4.** Is your diarrhea:	BETTER	**WORSE**	SAME
**5.** Is the blood in your stool:	BETTER	**WORSE**	SAME
**6.** Is there a presence of a new rash on your body?	**YES**		NO

DESCRIPTION OF SYMPTOMS THAT HAVE WORSENED:

NAME OF PHYSICIAN INFORMED IF PATIENT RESPONSE CORRESPONDS TO ANY OF THE BOLDED ITEMS ABOVE:

________________________MD/DO

PHYSICIAN DECISION OF FURTHER EVALUATION:

CLINIC	EMERGENCY ROOM	PHONE FOLLOW-UP

APPENDIX F1 (Ulcerative Colitis or Indeterminate Colitis):

FOLLOW UP PHONE CALL/ADVERSE EVENT: CASE REPORT FORM

WEEK(S)/MONTH(S) POST-TRANSPLANT ___

RECIPIENT CODED No.: __________________

DATE/TIME OF CALL: ___ ___/___ ___/___ ___ ___:___AM/PM

<table>
<tr><td>1. How do you feel today?</td><td colspan="2">WELL</td><td colspan="2">UNWELL</td></tr>
<tr><td>2. Have you had a fever > 100.4 degrees Celsius since the last phone call?</td><td colspan="2">YES</td><td colspan="2">NO</td></tr>
<tr><td>3. Has your abdominal pain:</td><td>RESOLVED</td><td>IMPROVED</td><td>WORSENED</td><td>N/A</td></tr>
<tr><td>4. Has your diarrhea:</td><td>RESOLVED</td><td>IMPROVED</td><td>WORSENED</td><td>N/A</td></tr>
<tr><td>5. Frequency of stools/day:</td><td><3</td><td colspan="2">3-6</td><td>>6</td></tr>
<tr><td>5. Has the blood in your stool:</td><td>RESOLVED</td><td>IMPROVED</td><td>WORSENED</td><td>N/A</td></tr>
<tr><td rowspan="2">6. Have you been fatigued?</td><td colspan="3">YES</td><td rowspan="2">NO</td></tr>
<tr><td>Mild</td><td>Moderate</td><td>Severe</td></tr>
<tr><td>7. Have you experienced weight changes since the last phone call?</td><td>NO</td><td colspan="2">YES</td><td>± ____ LBS</td></tr>
<tr><td rowspan="2">8. Have you required antibiotics since the fecal transplant? If yes, what is the name of the antibiotic(s)?</td><td colspan="2">YES</td><td colspan="2" rowspan="2">NO</td></tr>
<tr><td colspan="2">Name of antibiotic(s):
_______________</td></tr>
<tr><td rowspan="2">9. Have you taken any new medication(s), including OTC or probiotics since the last phone call?</td><td colspan="2">YES</td><td colspan="2" rowspan="2">NO</td></tr>
<tr><td colspan="2">Name of medication(s):
_______________</td></tr>
<tr><td rowspan="2">10. Have you developed any NEW medical conditions since the last phone call?</td><td colspan="2">YES</td><td colspan="2" rowspan="2">NO</td></tr>
<tr><td colspan="2">Please specify:
_______________</td></tr>
<tr><td rowspan="2">11. Did any medical condition(s) you had before your fecal transplant go away after your fecal transplant?</td><td colspan="2">YES</td><td colspan="2" rowspan="2">NO</td></tr>
<tr><td colspan="2">Please specify:
_______________</td></tr>
<tr><td>11. Please list all medication(s) you take on a regular basis (dosage not necessary) including</td><td colspan="4">Name of medication(s):
_______________________________________</td></tr>
</table>

chemotherapeutic agents, if applicable	

DESCRIPTION OF ADVERSE EVENT(S):

(An adverse event will be defined as any unfavorable or unintended sign, symptoms, disea1se, syndrome, abnormal laboratory finding, or concurrent illness that emerges or worsens relative to the recipient's pre-transplant baseline, whether or not it is considered to be related to the fecal transplantation)
All Grade 3 and 4 adverse events will be sent to the Data Safety Monitoring Board.

NAME OF PHYSICIAN INFORMED IF PATIENT RESPONSE CORRESPONDS TO ANY OF THE BOLDED ITEMS ABOVE:

________________________MD/DO

PHYSICIAN DECISION OF FURTHER EVALUATION:

CLINIC	EMERGENCY ROOM	PHONE FOLLOW-UP

DOCUMENTATION SENT TO DATA SAFETY MONITORING COMMITTEE ON ___ ___/___ ___/___ ___:

YES	NO

APPENDIX F2 (*Clostridium difficile*):

FOLLOW UP PHONE CALL/ADVERSE EVENT: CASE REPORT FORM

WEEK(S)/MONTH(S) POST-TRANSPLANT ___

RECIPIENT CODED No.: ___________________

DATE/TIME OF CALL: ___ ___/___ ___/___ ___ ___:___AM/PM

**1.** How do you feel today?	WELL		**UNWELL**	
**2.** Have you had a fever > 100.4 degrees Celsius since the last phone call?	**YES**		NO	
**3.** Has your abdominal pain:	RESOLVED	IMPROVED	**WORSENED**	N/A
**4.** Has your diarrhea:	RESOLVED	IMPROVED	**WORSENED**	N/A
**5.** Frequency of stools/day:	<3	3-6	**>6**	
**5.** Has the blood in your stool:	RESOLVED	IMPROVED	**WORSENED**	N/A
**6.** Have you been fatigued?	**YES**			NO
	**Mild**	**Moderate**	**Severe**	
**7.** Have you experienced weight changes since the last phone call?	NO	**YES**	± ____ LBS	
**8.** Have you required antibiotics since the fecal transplant? If yes, what is the name of the antibiotic(s)?	YES		NO	
	Name of antibiotic(s): _______________			
**9.** Have you taken any new medication(s), including OTC or probiotics since the last phone call?	YES		NO	
	Name of medication(s): _______________			
**10.** Have you developed any NEW medical conditions since the last phone call?	**YES**		NO	
	**Please specify: _______________**			
**11.** Did any medical condition(s) you had before your fecal transplant go away after your fecal transplant?	YES		NO	
	Please specify: _______________			
**11.** Please list all medication(s) you take on a regular basis (dosage not necessary) including	Name of medication(s): ____			

chemotherapeutic agents, if applicable	

DESCRIPTION OF ADVERSE EVENT(S):

(An adverse event will be defined as any unfavorable or unintended sign, symptoms, disease, syndrome, abnormal laboratory finding, or concurrent illness that emerges or worsens relative to the recipient's pre-transplant baseline, whether or not it is considered to be related to the fecal transplantation)
All Grade 3 and 4 adverse events will be sent to the Data Safety Monitoring Board.

NAME OF PHYSICIAN INFORMED IF PATIENT RESPONSE CORRESPONDS TO ANY OF THE BOLDED ITEMS ABOVE:

________________________MD/DO

PHYSICIAN DECISION OF FURTHER EVALUATION:

CLINIC	EMERGENCY ROOM	PHONE FOLLOW-UP

FOLLOW UP *C. DIFFICILE* TESTING<br>DATE OF TESTING: __ __/__ __/__ __	POSITIVE	NEGATIVE

DOCUMENTATION SENT TO DATA SAFETY MONITORING COMMITTEE ON ___ ___/___ ___/___ ___:

YES	NO

APPENDIX G:

SERIOUS ADVERSE EVENT (SAE): CASE REPORT FORM

WEEK(S)/MONTH(S) POST-TRANSPLANT ___

☐ INITIAL REPORT/☐ FOLLOW-UP REPORT

RECIPIENT CODED No.: ________________

ONSET OF EVENT: ___ ___/___ ___/___ ___

Severity of SAE	MILD	MODERATE	SEVERE

Outcome of SAE:	☐ RECOVERED WITH SEQUELAE	RECOVERY DATE ___ ___/___ ___/___ ___
	☐ RECOVERED WITHOUT SEQUELAE	
	☐ PERSISTING	☐ UNKNOWN/LOST TO FOLLOW-UP
	☐ DEATH	

SAE category:	☐ DEATH*	☐ LIFE THREATENING
	☐ HOSPITALIZATION REQUIRED#	☐ PROLONGED IN-PATIENT HOSPITALIZATION#
	☐ PERSISTENT/SIGNIFICANT DISABILITY OR INCAPACITY	☐ OTHER MEDICALLY IMPORTANT CONDITION

*IF DEATH HAS OCCURRED:

**1.** Date of death	___ ___/___ ___/___ ___
**2.** Primary cause of death (if known)	____________________

#IF RECIPIENT HAS REQUIRED HOSPITALIZATION:

**1.** Hospital admission date	___ ___/___ ___/___ ___
**2.** Current duration of hospitalization	__________ (WEEKS/MONTHS)

DESCRIPTION OF SERIOUS ADVERSE EVENT(S):

NAME OF PHYSICIAN INFORMED OF SAE:
________________________MD/DO

DOCUMENTATION SENT TO DATA SAFETY MONITORING COMMITTEE ON ___ ___/___ ___/___ ___:

YES	NO

APPENDIX H:

Pediatric Ulcerative Colitis Activity Index

WEEK(S)/MONTH(S) POST-TRANSPLANT ___

RECIPIENT CODED No.: ____________________

Item	Points
**1. Abdominal pain**	
No pain	0
Pain can be ignored	5
Pain cannot be ignored	10
**2. Rectal bleeding**	
None	0
Small amount only, in <50% of stools	10
Small amount with most stools	20
Large amount (>50% of stool content)	30
**3. Stool consistency of most stools**	
Formed	0
Partially formed	5
Completely formed	10
**4. Number of stools per 24 hours**	
0-2	0
3-5	5
6-8	10
>8	15
**5. Nocturnal stools (any episode causing wakening)**	
No	0
Yes	10
**6. Activity level**	
No limitation of activity	0
Occasional limitation of activity	5
Severe restricted activity	10
**Total Score**	_________

APPENDIX I:

Mayo Score

WEEK(S)/MONTH(S) POST-TRANSPLANT ___

RECIPIENT CODED No.: ___________________

Item	Points
**1. Stool Frequency**	
Normal number of stools for the patient	0
1-2 stools more than normal	1
3-4 stools more than normal	2
5 or more stools more than normal	3
**2. Rectal bleeding**	
None	0
Streaks of blood with stool less than half of the time	1
Obvious blood with stool most of the time	2
Blood alone passed	3
**3. Endoscopic Findings**	
Normal or inactive disease	0
Mild disease (erythema, decreased vascular pattern, mild friability)	1
Moderate disease (marked erythema, absent vascular pattern, friability, erosions)	2
Severe disease (spontaneous bleeding, ulceration)	3
**4. Physician's Global Assessment**	
Normal	0
Mild disease	1
Moderate disease	2
Severe disease	3
**Total Score**	_________

APPENDIX J:

Study Flowsheet

<table>
<tr><th>Protocol</th><th>Day(s)</th><th colspan="2"></th></tr>
<tr><td rowspan="2">Recruitment of stool recipient
(Appendix A1/A2)</td><td rowspan="2">-14</td><td rowspan="2">Performed?</td><td>Yes</td></tr>
<tr><td>No</td></tr>
<tr><td rowspan="2">Donor screening
(Appendix B)</td><td rowspan="2">-14</td><td rowspan="2">Eligible?</td><td>Yes</td></tr>
<tr><td>No</td></tr>
<tr><td rowspan="2">Donor Serum and Stool Screening</td><td rowspan="2">-7</td><td rowspan="2">Eligible?</td><td>Yes</td></tr>
<tr><td>No</td></tr>
<tr><td>Diet Record completed by Recipient?
(Appendix C)</td><td rowspan="2">-7 to 0</td><td>Yes</td><td>No</td></tr>
<tr><td>Diet Record completed by Donor?</td><td>Yes</td><td>No</td></tr>
<tr><td>Pre-transplant stool collection?*</td><td>-3 to -1</td><td>Yes</td><td>No</td></tr>
<tr><td>Recipient undergoing bowel prep with
resultant clear yellow stool?</td><td>-1</td><td>Yes</td><td>No</td></tr>
<tr><td>Donor stool processed?
(Appendix D)</td><td>0</td><td>Yes</td><td>No</td></tr>
<tr><td>Day of transplant stool collection?*</td><td>0</td><td>Yes</td><td>No</td></tr>
<tr><td>Recipient blood and/or saliva obtained? *</td><td>0</td><td>Yes</td><td>No</td></tr>
<tr><td>Patient monitored?</td><td>0</td><td>Yes</td><td>No</td></tr>
<tr><td>Follow-up Phone Call? (Appendix E)</td><td>1</td><td>Yes</td><td>No</td></tr>
<tr><th>Protocol</th><th>Week (s)</th><th colspan="2"></th></tr>
<tr><td>Follow-up Phone Call? (Appendix F)</td><td rowspan="2">1</td><td>Yes</td><td>No</td></tr>
<tr><td>Post-transplant stool collection?*</td><td>Yes</td><td>No</td></tr>
<tr><td rowspan="2">Follow-up Phone Call? (Appendix F)</td><td>2</td><td>Yes</td><td>No</td></tr>
<tr><td>3</td><td>Yes</td><td>No</td></tr>
<tr><td>Follow-up Clinic Visit?
(Appendix F) and (Appendix H or I)</td><td>4</td><td>Yes</td><td>No</td></tr>
<tr><td rowspan="7">Follow-up Phone Call? (Appendix F)</td><td>5</td><td>Yes</td><td>No</td></tr>
<tr><td>6</td><td>Yes</td><td>No</td></tr>
<tr><td>7</td><td>Yes</td><td>No</td></tr>
<tr><td>8</td><td>Yes</td><td>No</td></tr>
<tr><td>9</td><td>Yes</td><td>No</td></tr>
<tr><td>10</td><td>Yes</td><td>No</td></tr>
<tr><td>11</td><td>Yes</td><td>No</td></tr>
<tr><td>Follow-up Clinic Visit?
(Appendix F) and (Appendix H or I)</td><td rowspan="2">12</td><td>Yes</td><td>No</td></tr>
<tr><td>Post-transplant stool collection?*</td><td>Yes</td><td>No</td></tr>
</table>

Protocol	Month (s)		
Follow-up Phone Call? (Appendix F)	4	Yes	No
	5	Yes	No
Follow-up Clinic Visit? (Appendix F) and (Appendix H or I)	6	Yes	No
Follow-up Phone Call? (Appendix F)	7	Yes	No
	8	Yes	No
Follow-up Clinic Visit? (Appendix F) and (Appendix H or I)	9	Yes	No
Follow-up Phone Call? (Appendix F)	10	Yes	No
	11	Yes	No
Follow-up Clinic Visit? (Appendix F) and (Appendix H or I)	12	Yes	No

APPENDIX K:

DIARY CARD

DATE:__________________

DESCRIPTION OF SYMPTOMS THAT ARE NEW OR WORSENED:

DATE:__________________

DESCRIPTION OF SYMPTOMS THAT ARE NEW OR WORSENED:

DATE:__________________

DESCRIPTION OF SYMPTOMS THAT ARE NEW OR WORSENED:

DATE:__________________

DESCRIPTION OF SYMPTOMS THAT ARE NEW OR WORSENED:
